# Intervention patterns and preliminary effectiveness on Social Participation following stroke: a scoping review

**DOI:** 10.1186/s12883-023-03250-2

**Published:** 2023-07-18

**Authors:** Xuan Zhou, Minxia Du, Xiaojie Dai, Shenghui Zhu, Lanshu Zhou, Xuemei Li

**Affiliations:** 1 Department of nursing, Naval Medical University, Shanghai, 200433 China; 2 Department of nursing, Xinxiang Medical University, Henan, 453000 China; 3 Department of Orthopaedics, Changzheng Hospital, Shanghai, 200003 China; 4 Department of nursing, Community Health Service Centre, Nanjing East Road, Huangpu District, Shanghai, 200002 China; 5 Nursing Department, Naval Medical University, 800 Xiangyin Road, Yangpu District, Shanghai, 200433 China

**Keywords:** Social participation, Stroke, Scoping review, Exercise-based intervention, Complex intervention, Self-management program, Occupational therapy

## Abstract

**Background:**

Stroke survivors suffer an overall loss of social participation. However, the interventions aiming at improving social participation have not yet been well-established. There is a need to synthesize existing knowledge on clinical interventions aiming at improving social participation among people with stroke.

**Objective:**

To describe the patterns of intervention that have been applied to stroke survivors to improve social participation and to determine the preliminary effects of these patterns.

**Methods:**

Eight online databases, including Cochrane Library, PubMed, Web of Science, Embase, Medline, CINAHL plus, PsycINFO, and Scoups were searched with predefined search terms from inception to September 22, 2022. References of included articles and previous reviews were also checked to identify additional studies. Two reviewers independently selected eligible studies and extracted data from the included articles.

**Results:**

A total of 98 studies were included, of which only 25 studies considered social participation as primary outcome of clinical interventions. The patterns of intervention were various, consisting of exercise-based intervention, occupational therapy, self-management program, and complex intervention. Of the 25 studies, eight studies found a positive effect of relative clinical intervention on social participation for stroke survivors. Of note, the same modality of intervention such as exercise-based intervention and self-management program produced paradoxical conclusion on social participation.

**Conclusion:**

Exercised-based intervention, occupational therapy, self-management program, and complex intervention were important intervention modalities for the improvement of social participation among stroke survivors. Even though the preliminary effectiveness on social participation seems to be potentially positive, further high-quality researches are still required to reach a consensus to achieve optimal social participation among stroke survivors.

**Supplementary Information:**

The online version contains supplementary material available at 10.1186/s12883-023-03250-2.

## Introduction

Stroke has been a major global health concern, characterized by high morbidity, and a high rate of disability and recurrence [1]. With the increased population aging worldwide, the significance of stroke appears to increase in the future [2]. The main problem faced by stroke survivors is the long-term impact of disability on physical, psychological, and social function. Early-stage and continuous rehabilitation have been considered as a major buffer against unexpected changes resulting from stroke [3].

Social participation that refers to involvement in the life situation has been viewed as an ultimate outcome of successful rehabilitation according to the International Classification of Functioning, Disability, and Health (ICF) [4, 5]. Despite a strong willingness of stroke survivors to return to their previous lifestyle, their social participation was severely impaired [6]. Current evidence has found that there were tremendous difficulties for stroke survivors to re-engage in work, valued activities, and interpersonal interaction [7, 8]. Stroke survivors with a low level of social participation are at increased risk for adverse health outcome, including low mood, recurrent stroke, low quality of life, and even mortality [9]. As a result, the improvement of social participation among stroke survivors has been a primary focus of community rehabilitation [10].

Post-stroke social participation is a major public concern, which is associated with stroke-related physical dysfunction (including limb function, communication barriers, and cognition), emotion disorders (such as depression and isolation), and environment [11, 12]. There is an urgent demand for effective intervention program to continuously enhance social participation among stroke survivors. However, current rehabilitation studies frequently did not include social participation as a routine measurement outcome [13]. Some clinical trials merely included social participation as a secondary outcome rather than a primary outcome. In fact, current interventions mainly focus on the effectiveness on stroke survivors’ physical function rather than social participation itself [14, 15]. Of note, a good physical function was not always associated with satisfactory social participation among stroke survivors. A previous study pointed out that although stroke survivors showed very mild impairment and disability, their participation in daily activities and social role was substantially impacted [16].

Collectively, social participation among stroke survivors remains unsatisfactory, and the interventions aiming at improving social participation are not well-established. Thus, it is necessary to improve knowledge about clinical interventions for the improvement of social participation among stroke survivors. A scoping review that can outline key concepts, types of evidence, and gaps in research related to specific areas is an effective form of knowledge synthesis [17, 18]. Scoping review provides guidance for future research priorities by systematically searching, selecting, and integrating existing knowledge. Therefore, the aim of this scoping review is to (1) map the current evidence surrounding the interventions for improving social participation among stroke survivors and identify knowledge gaps in the researches, and (2) determine the preliminary effectiveness of the interventions on social participation among stroke survivors. This scoping review is expected to provide a basis for follow-up interventional studies.

## Methods

This scoping review was conducted based on the methodology outlined by Arksey and O’ Malley and refined by Levac et al. and the PRISMA Extension for Scoping Reviews (PRISMA-ScR) [17, 18]. The methodology mainly involved six stages, (1) identifying the research questions; (2) searching related studies; (3) screening the studies; (4) charting the data; (5) collating, summarizing, and reporting the results; and (6) consultation with broader stakeholder, respectively. A scoping review methodology was selected because it can address more broad questions and identify the types of available evidence in a given field [19]. A scoping review also serves as a precursor to a systematic review [19]. This methodology assists to identify the gaps in current knowledge base to provide guide for further research in the field. The scoping review hasn’t been registered anywhere.

### Search strategy

Eight online databases, including Cochrane Library, PubMed, Web of Science, Embase, Medline, CINAHL plus, PsycINFO, and Scoups were searched from database inception to September 22, 2022 to hit relevant studies concerning interventions to improve social participation among stroke survivors. The search terms were “stroke” and “participation”, and synonyms and Boolean operators were used in the search strategy. The specific search strategy was showed in Additional file (Supplement Table 1). There was no restriction on language published and study design. Additionally, a hand search was performed to select the references of included studies and previously published reviews.

### Study selection

**Fig. 1:**
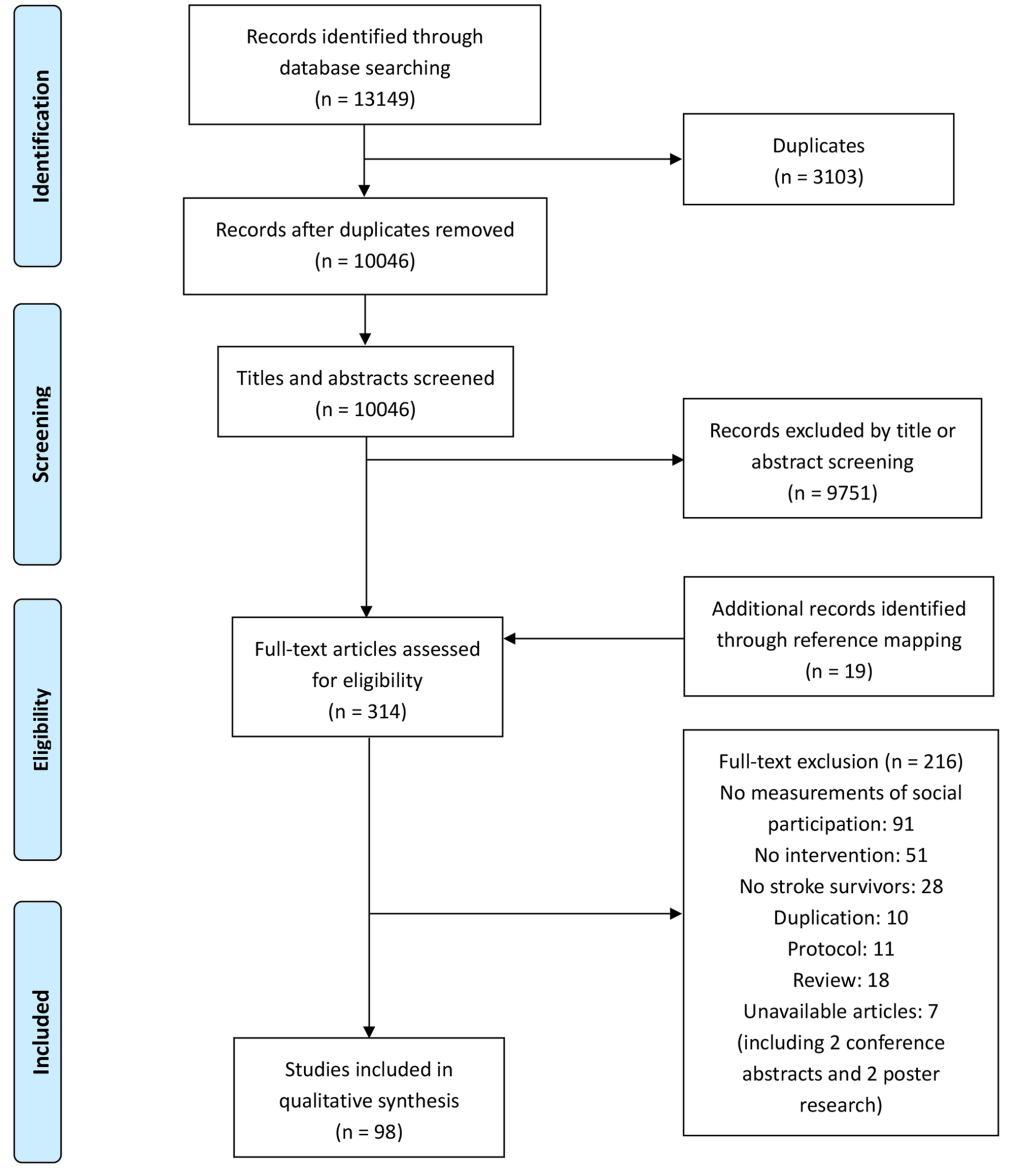
Reference screening flow chart

A total of 13,149 searched studies from the 8 databases were imported into Endnote X9 to exclude duplicates (Fig. 1). The inclusion criteria for studies in this scoping review were: (1) study participants who were stroke survivors and aged over 18 years; (2) experimental study; (3) outcome measures including social participation; and (4) social participation outcome evaluated using scales but not the qualitative description. The exclusion criteria were (1) participants with severe comorbidities such as malignant tumors and organ dysfunction; (2) animal experiments; and (3) unavailable full text. Additionally, study reviews were excluded, but their references were checked for presence in the search result. Two review authors independently screened titles, abstracts, and full-text articles based on inclusion and exclusion criteria after training, with a third review author available to moderate any disagreements. The review authors also contacted the investigators for additional data when required.

### Quality appraisal

Cochrane risk of bias tool in the Cochrane Handbook (version 5.1.0) was used for the randomized controlled trials (RCTs) [44]. A total of seven indicators (random sequence generation, allocation concealment, blindness of participant and personnel, blindness of outcome assessment, incomplete outcome data, selective reporting, and other bias) was evaluated using “low risk”, “unclear risk”, or “high risk”. The criteria of the Australian Evidence-based Health Care Center for non-randomized controlled trials (non-RCTs) were used as well [45]. Each entry was assessed with “yes”, “no”, “unclear”, or “not applicable”. The quality appraisal was performed only for studies that considered social participation as primary outcome measure.

### Data extraction

Data extraction was independently performed by two authors using Excel 2016 for tabulating the descriptive data from all included studies. The detailed information included author, year, country, participant characteristics (time from onset, and age), study design, sample size, study target, intervention, setting, training dosage, measurement point, the type of social participation outcome (primary or secondary), measurement tool of social participation, and results. Any disagreements between reviewers were resolved by consensus through team discussion.

### Narrative synthesis

Narrative analyses were performed to summarize the study results. results. The analyses were guided by a three-step narrative synthesis framework: (1) develop a preliminary synthesis; (2) explore relationship between data; and (3) assess the robustness of the synthesis [46]. The first step was performed by identifying patterns using tabulation, grouping and thematic analysis. The second step was conducted by analyzing participant demographics, measurement of social participation, and intervention characteristics. Finally, the robustness of the synthesis was represented by methodological quality of included studies that considered social participation as primary outcome, the information required from included studies, and strength of conclusion that could be made. Additionally, frequencies and percentages were calculated to summarize the data if necessary.

## Results

A total of 98 studies selected were included in the review. According to the primary outcome or secondary outcome of social participation, the data extracted were presented in two parts. The summary of 25 included studies where social participation was considered as primary outcome is shown in Tables 1 and 2. 73 studies where social participation was considered as secondary outcome were reported in Additional file (Supplementary Tables 2 and Table 3). All 98 published articles were distributed within a time frame of 20 years (2001–2022). Average two or three articles per year were published relevant to interventions to improve poststroke social participation from 2001 to 2012. However, since 2013, a substantial increase in the number of studies related to our scoping purpose. There were 72 studies published between 2013 and 2022, accounting for 73.5%. Of the 25 studies, four studies were from America, followed by Canada (N = 3), Sweden (N = 3), and the Netherland (N = 3). The majority of study designs were RCTs (76.0%), and the remaining studies were pre-post clinical trials. Of the 73 studies, 16 studies were from Canada (21.9%), 12 studies from America (16.4%), eight studies from Australia (11.0%), eight studies from China (11.0%), and seven studies from the UK (9.6%), respectively. The RCTs accounted for 68.5%. The other studies were single-group pre-post design and control studies (Supplementary Table 2).

**Table 1:** Basic Characteristics of Included Studies

Studies	Year	Country	Study Design	Sample size (T/C)	Age [M (SD) or Median (range/IQR)]	Time after onset	Comorbidities	Measurement Time	Measurement Tool	Operational definition
de Rooij et al. [20]	2021	Netherlands	RCT	52 (28/24)	T: 65; C: 61	from 2 weeks to 6 months	Ability to walk without physical assistance	T1, T2, T3 (3 months post intervention)	USER-P	Participation
Tarrant et al. [21]	2021	UK	RCT	41 (20/21)	T: 65.2 (12.2); C: 67.7 (8.3)	Not reported	Aphasia	T1, T2 (3 months post-randomization), T3 (6 months post-randomization)	RNLI	Social participation
Bin Zainal et al. [22]	2020	Singapore	Retrospective pre-post-test design	50	44 (38, 48)	Not reported	Not clear	T1, T3	CIQ	Community reintegration
Harel-Katz et al. [23]	2020	Israel	RCT	39 (20/19)	T: 65 (52, 75); C: 66 (45, 87)	<1.5 years	Mild physical impairment	T1, T2	RNLI	Participation
Cruice et al. [24]	2020	UK	Pre-post-test design	27	61.3 (11.1)	≥ 4 months	Aphasia	T1, T2, T3 (8 weeks post intervention)	SNA; ALA	Social participation
Chinchai et al. [25]	2020	Thailand	Pre-post-test design	25	31–40 years: 3; 41–50 years: 5; 51–60 years: 5; >60 years: 12	Not reported	Not clear	T1, T2	CIQ	Community integration
Hedman et al. [26]	2018	Sweden	RCT	145 (71/74)	T: 71 (9); C: 68 (9)	<3 months	Not clear	T1, T3 (5 years post intervention)	SIS-P; OGQ	Perceived participation
Aramaki et al. [27]	2019	Brasil	Pre-post-test design	10	41.3 (12.77)	≤ 5years	Hemiparesis	T1, T2	Participation Scale	Social participation
Strk et al. [28]	2017	USA	RCT	15 (9/6)	T: 66.89 (7.96); C: 64.67 (8.21)	Not reported	No obvious complications	T1, T3 (6 months and 12 months post intervention)	RNLI	Community participation
Kamwesiga et al. [29]	2018	Sweden	Pre-post-test design	28 (13/15)	T: 61.2 (15.0); C: 58.5 (14.0)	Not reported	Disability	T1, T2	SIS-P	Participation
Brouwer et al. [30]	2018	Canada	RCT	103 (51/52)	T: 62.7 (1.9); C: 62.1 (1.8)	Not reported	Lower limb function impairment	Discharge and 3 months, 6 months, 9 months, 12 months, and 15 months after discharge	SIPSO	Community reintegration
Van de Ven et al. [31]	2017	Netherlands	RCT	97 (38/35/24)	T: 57.0 (9.1); C1: 60.9 (7.5); C2: 61.2 (9.0)	3 months to 5 years	Cognitive impairment	T1, Middle intervention, T2, T3 (4 weeks post intervention)	USER-P	Participation in society
Kessler et al. [32]	2017	Canada	RCT	21 (10/11)	T: 71.0 (13.2); C: 64.9 (16.3)	Not reported	Not clear	T1, T2, T3 (6 months post intervention)	RNLI	Participation
Wang et al. [33]	2014	China	RCT	51 (25/26)	T: 62.0 (9.5); C: 65.4 (10.6)	> 6 months	Mild to moderate disability	T1, T2	SIS-P	Social participation
Tielemans et al. [34]	2015	Netherlands	RCT	113 (58/55)	T: 55.2 (8.9); C: 58.8 (8.7)	≥ 6 weeks	Cognitively impaired (partly); Communicatively impaired (partly)	T1, T2, T3 (3 months and 9 months post intervention)	USER-P	Participation restrictions
Mckellar et al. [35]	2015	Canada	RCT	77 (39/38)	T: 57.70 (29, 78); C:60.22 (25, 85)	Not reported	Not clear	T2	RNLI	Reintegration into social activities
Guidetti et al. [36]	2015	Sweden	RCT	280 (129/151)	T: 74 (10); C: 71 (11)	≤ 3 months	Not clear	T1, 3, 6 and 12 months after inclusion	SIS-P; FAI; OGQ; IPA	Perceived participation
Muller et al. [37]	2014	America	RCT	13	46	Not reported	Not clear	T1, T2	CIQ	Social integration
Kim et al. [38]	2014	Korea	RCT	22 (11/11)	T: 50.18 (10.29); C: 50.73 (7.24)	> 6 months	Lower limb function impairment	T1, T2	SIS-P	Social Participation
Marsden et al. [39]	2010	Australia	RCT	25 (12/13)	T: 70.0 (9.0); C: 73.1 (9.3)	> 4 weeks	Mild to moderate disability	T1, T2 (9 weeks weeks after entry, T3 (21 weeks after entry)	SIS-P	Participation
Harrington et al. [40]	2010	England	RCT	243 (119/124)	T: 71 (10.5); C: 70 (10.2)	> 3 months	Not clear	T1, T2, T3 (6 months and 12 months post intervention)	SIPSO	Social integration
Smith et al. [41]	2008	America	RCT	20 (10/10)	T: 57.8 (7.0); C: 56 (8.3)	3month to 2 years	Lower extremity motor impairment	T1, T2, T3 (6 weeks post intervention)	SIS-P	Social participation
Katz-Leurer et al. [42]	2003	Israel	RCT	92 (46/46)	63 (11)	> 48 h	Not clear	T1, T2	Subscale of FAI	Social outings
Parker et al. [42]	2001	England	RCT	466 (153/156/157)	T1: 70 (65, 79); T2: 71 (66, 78); C: 72 (65, 78)	< 6 month	Not clear	T1, T2, T3 (12 months post intervention)	NLQ	Leisure activity
Hinckley et al. [43]	2001	America	Pre-post-test design	36 (21/15)	T: 56.2; C: 62	Not reported	Aphasia	T1, T2	CIQ	Social integration

Note: C, Control group; T, Trail group; RCT, Randomized Controlled Trial; T1, Baseline; T2, Post-intervention; T3, Follow-up; ALA: The Assessment for Living with Aphasia; CIQ, The Community Integration Questionnaire; FAI, The Frenchay Activity Index; IPA, The Impact on Participation and Autonomy Questionnaire; NLQ, Nottingham Leisure Questionnaire; OGQ, Occupational Gaps Questionnaire; RNLI, Reintegration to Normal Living Index; SIPSO, Subjective Index of Physical and Social Outcome; SIS-P, Participation subscale of Stroke Impact Scale; SNA, Social Network Assessment; USER-P, Utrecht Scale for Evaluation of Rehabilitation-Participation

**Table 2:** Intervention Information of Included Studies

Studies	Setting	Dosage	Experiment Group	Control Group	Results
de Rooij et al. [20]	Rehabilitation center	12 30-minute sessions during 6 weeks	Virtual reality gait training (VRT)	Treadmill training and functional gait exercises without virtual reality	Negative
Tarrant et al. [21]	Community facility	10 weekly singing group sessions	Aphasia information resource pack and singing intervention	Aphasia information resource pack	Negative
Bin Zainal et al. [22]	Community-based voluntary welfare organization.	Not reported	A pilot community-based interdisciplinary vocational rehabilitation program: physical rehabilitation, psychosocial support, employment support, and caregiver support services	NA	Positive
Harel-Katz et al. [23]	Community day-rehabilitation center	12 weekly group sessions, each lasting 2.5 h	Improving Participation After Stroke Self-Management program (IPASS) and standard individual therapy or standard care	Standard individual therapy or standard care only	Negative
Cruice et al. [24]	Home	Two one-hour sessions per week for 8 weeks	Supported conversation provided over Skype (telerehabilitation)	NA	Positive
Chinchai et al. [25]	Community rehabilitation centers	Twice a week, one and a half hours each time, for a period of three months	Occupational therapy programs: rehabilitation by Village Health Volunteers	NA	Positive
Hedman et al. [26]	Inpatient or home-based rehabilitation units	The number of occupational therapy sessions was not limited or decided in advance for either group	Client-centered activities of daily living (ADL) intervention	Usual ADL interventions	Negative
Aramaki et al. [27]	Rehabilitation Center	40 min per day, three days per week, for 12 weeks	Client-centered virtual reality	NA	Negative
Strk et al. [28]	Home	One pre-discharge and five post-discharge home visits	Home modification and community participation intervention	Evidence-based stroke education program	Not tested
Kamwesiga et al. [29]	Home	Eight-week intervention	A mobile phone supported family-centered intervention (F@ce™)	Did not receive F@ce™ intervention	Negative
Brouwer et al. [30]	Three research laboratories	1-hour session, 3 times per week for 2 weeks	Client-centered rehabilitation intervention (tune-up): 5 key elements (strength/power, balance, cardiovascular endurance, motor coordination, and education about relevant community resources, as well as how to monitor their mobility and activities)	NA	Negative
Van de Ven et al. [31]	Home	Five times per week and a total of 58 half-an-hour sessions across 12 weeks	Computer-based training programs	Group 1: active control: mock training Group 2: waiting list control	Negative
Kessler et al. [32]	Home	10 sessions over 16 weeks	Occupational Performance Coaching (OPC) and usual care	Usual care	Not tested
Wang et al. [33]	Home	12 weeks (at least 60- to 90-minute sessions)	Caregiver-mediated, home-based intervention (CHI): phase 1, to improve patients’ body functions and structural components; phase 2, to improve patients’ ability to undertake everyday activities; and phase 3, to help the patients reintegrate into the society	Received visits from the therapist without intervention	Positive
Tielemans et al. [34]	Outpatient facilities	T: seven 2-hour sessions in 10 weeks C: four 1-hour sessions in 10 weeks	A self-management intervention	Education intervention	Negative
Mckellar et al. [35]	Not reported	T: two 20-minute visit; C: a brief in-person visit	The Community Re-engagement Cue to Action Trigger Tool (CRCATT)	Received only the Heart and Stroke Foundation booklet during a brief in-person visit	Negative
Guidetti et al. [36]	Sixteen rehabilitation units	Not determined in advance: T: 71 (7 to 269) days; C: 59 (1 to 402) days	A client-centred ADL intervention	Usual ADL intervention	Negative
Muller et al. [37]	Hospital	90-minute twice a month over 18-week period	Hospital-based program based on occupational therapy principles: active engagement and client-centered educational topics	NA	Positive
Kim et al. [38]	Rehabilitation ward	CWTP: 30 min per day, five times a week, for four weeks; Standard rehabilitation program: 60 min per day, five times a week, for four weeks	Community Walking Training Program (CWTP) and standard rehabilitation program consisting of physical and occupational therapy	Standard rehabilitation program consisting of physical and occupational therapy	Positive
Marsden et al. [39]	Rural outpatient	Once per week over seven weeks	Yoga intervention	No intervention	Positive
Harrington et al. [40]	Leisure and community centers	Twice a week, 16 2-hour sessions across 8 weeks	Community-based exercise and education scheme for stroke	Standard care and an information sheet detailing local groups and contact numbers	Short-term result: negative Long-term result: positive
Smith et al. [41]	Outpatient stroke center	12 sessions across 4week, 20 min each session	Treadmill training	Follow-up, rehabilitation log	Positive
Katz-Leurer et al. [47]	Rehabilitation unit	Phase 1: 2 to10 minute train, 5 days per week for two weeks; Phase 2: 30-minute train, three times a week for six weeks	Trained on a leg cycle ergometer and regular therapy	Regular therapy	Negative
Parker et al. [42]	Home	Ten more than 30 min-sessions over 6 months	T1: leisure therapy; T2: ADL group	No occupational therapy	Negative
Hinckley et al. [43]	Home	Two-day education program	Family education seminars	Not participating	Negative

### Quality appraisal

The quality appraisal of studies considering social participation as primary outcome is represented in Additional file (Supplementary Tables 4 and Table 5). For RCTs, the leading risk was the blindness of participant and personnel. While many studies complied with the blindness of outcome assessment, the assessors might become aware of the allocation through patients’ responses during interviews, which may result in some bias. The randomization was generally performed. Allocation concealment in some studies was high risk or unclear risk due to lack of specific description. The overall study quality for RCTs was medium quality and above. For non-RCTs, the main risk was lack of control group and detailed description of the incomplete follow-up. The methodological quality for non-RCTs was acceptable.

### Participants

Across the 25 articles, there were a total of 2091 participants. The average age or median age of the participants in most studies was over 60. There were only four articles with the mean age of the participants less than 50 [22, 25, 27, 37]. The period from onset to recruitment was various, which ranged from just 48 h after onset to several years following stroke. Three months and six months post-stroke were the frequently used time cutoff. With regards to the comorbidities, the participants in eight studies had physical impairments, including upper or limb function impairments, disability, and hemiparesis. Three articles reported post-stroke aphasia and two described cognitive impairments. The remaining studies had no clear description of the participants’ comorbidities. The results related to participants’ characteristics from 73 studies in which social participation was viewed as secondary outcome were similar with above description (Supplementary Table 2).

### Participation measures

The included studies covered a variety of operational definitions, as presented in Table 1. The term “participation” and “social participation” were the most common. Of the enrolled studies, a total of 12 measurement tools were used to assess social participation. The participation subscale of the Stroke Impact Scale (SIS) was the most frequently used tool by 7 of 25 studies (28.0%). The second most common tool was the Reintegration to Normal Living Index (RNLI, 20.0%), followed by the Community Integration Questionnaire (CIQ, 16.0%) and Utrecht Scale for Evaluation of Rehabilitation-Participation (USER-P, 12.0%). Of the 25 studies, 10 studies measured outcome at three time points (baseline, post-intervention, and follow-up) and 9 studies at two time points (baseline and post-intervention). When it comes to the follow-up measure, 3 months after the intervention was the most frequently used. Of the 73 included studies, the most frequently used tool was the participation subscale of the Stroke Impact Scale (SIS) as well. The most frequent selection of follow-up duration was 3 months after the intervention, followed by 6 months, one year, and one month after the intervention (Supplementary Table 2).

### Training dosage

The period of training varied from two days to six months. Specifically, 8 weeks (N = 4) and 12 weeks (N = 4) were the most frequently reported. The total sessions of the interventions varied from 2 sessions to 58 sessions, of which the top two sessions of intervention were 12 sessions (N = 3) and 10 sessions (N = 3). The session duration lasted from 10 min to 2.5 h, and the most frequent duration used was 30 min (N = 6), followed by 1 h (N = 2) and 2 h (N = 2). The session frequency ranged from twice a month to five times per week, and the most frequently reported session frequency was 2 to 3 times per week (N = 8). The different dosage for each study was presented detailly in Table 2. Of the 73 included studies, the most frequently used training period was 6–8 weeks. The top three sessions of intervention were 20 sessions (N = 10), 12 sessions (N = 7), and 36 sessions (N = 7), respectively. One-to-two-hour duration per session and 2 to 3 times per week were the most prevalent respectively (Supplementary Table 3).

### Training protocol

Of the 25 studies, eight studies focused on physical exercise. Specifically, two studies were dedicated to identifying the effect of virtual reality program on assisting in the functional recovery [20, 27]. The participants needed to perform games that were conducted difficultly in the initial evaluation using the Canadian Occupational Performance Measure (COPM). The games trained participants’ motor skills, motor coordination, and cognitive skills. The other two studies demonstrated the effectiveness of treadmill and leg cycle ergometer as assisted exercise devices on lower-limb functions [41, 47]. The remaining four studies described different exercise methods, such as walk training, yoga training, and strength, balance, and motor coordination trainings supported by Physiotherapists [33,41–43,,]. Additionally, post-stroke aphasia and cognitive impairment is two potential factors to influence social participation. Two studies examined respectively whether grouping singing and supported conversation provided over Skype could improve stroke survivors’ social participation [21, 24]. One article investigated the effectiveness of computer-based cognitive flexibility training [31]. Generally, close to half of the studies considered physical exercise as a significant intervention modality to promote social participation.

In addition to exercise-based intervention, occupational therapy was another frequently reported intervention for seven times [25, 26, 28, 29, 32, 36, 42]. During the treatment process, occupational therapists made an initial evaluation to clarify the participants’ ability by using COPM and helped them set appropriate goals for activities. Thereafter, the participants and occupational therapists together identified specific strategies that would enable successful performance of the chosen activity. The strategies consisted of problem solving, implementing new ways of performing activities of daily living (ADL), home modification, motivational enhancement, and self-management strategies. Some studies encouraged family to participate in occupational therapy [29]. It is possible to use mobile phones to supervise participants to perform activities themselves during occupational therapists’ follow-up intervals [29].

Self-management programs which focus on teaching stroke survivors’ important skills to manage their functional and emotional conditions, such as setting goals, problem-solving, decision-making, and resource utilization are becoming more prevalent, given the positive effect of self-management programs in other chronic diseases, such as diabetes, asthma, and chronic obstructive pulmonary disease. Two studies evaluated the feasibility and effectiveness of self-management strategies for reinforcement of social participation after stroke [23, 34]. Moreover, there were five studies that considered social participation as secondary outcome exploring the effectiveness of self-management interventions on helping stroke survivors to engage in the society. However, the studies indicated that the effectiveness of self-management interventions was controversial among stroke survivors. It was believed that one of the mechanisms accountable for the enhancement of health behaviors by self-management was strengthening individuals’ self-efficacy to manage their condition.

Health education was as well used to improve social participation after stroke in two studies [37, 43]. The education topics were various, including emotional support, communication strategies, driving, working, active strategies for coping, sleep, yoga. Additionally, the Community Re-engagement Cue to Action Trigger Tool (CRCATT), a patient-mediated question prompt list, covering eight areas was used to cue and supervise stroke survivors for re-engagement in activities post-stroke [35]. It was perceived that CRCATT could assisted stroke patients to play a more self-directed role by asking relevant questions and anticipating their needs. Complex intervention was the last reported intervention method. Generally, complex intervention contained different contents, such as physical exercise, emotional support, and strategy acquisition [22, 33]. For example, a caregiver-mediated, home-based intervention possessed the following functions:, to improve stroke patients’ body functions and structural components, to enhance patients’ ability to perform daily activities within their living environments using compensatory training methods, and to assist the patients return to the society by the participation in outdoor leisure activities [33].

Of the 73 studies with social participation as secondary outcome, exercise-based intervention was still the most frequently described (N = 40; Supplementary Table 3). However, the content of exercise-based intervention varied more widely compared with studies with social participation as primary outcome, which embodied body parts, assisted devices, settings, patients’ course of disease. It is the best of our acknowledged that common dysfunction after stroke includes motor dysfunction, cognitive dysfunction, speech dysfunction, swallowing dysfunction, sensory dysfunction and so on. Thus, the target of exercise-based intervention was diverse. Besides, the physical exercise was inseparable from advanced medical devices. For example, robot-based exercise was more prevalent in during the rehabilitation process post stroke. The training settings were not limited to hospitals and rehabilitation facilities, but extended to communities and participant’s homes. Short message and mobile calls quickly became increasingly receptive to urge the patients’ continuous training within their own living environment. Additionally, some studies combine cognitive training and aerobic exercise together in order to enhance the effectiveness of physical recovery. Similarly, occupational therapy was of great concern in improving social participation (N = 10). Occupational therapists were confronted with a change in task-targeted activities from ADL into social activities, such as meeting friends. Self-management strategies acquisition seemed to be as important as physical recovery with reported for five times. Complex intervention that referred multidisciplinary cooperation and multidimensional support was becoming increasingly prevalent among with reported for seven times. In addition, some intervention programs such as information providing, calls, stroke navigation service, consultation supported by nurses, health education were as well investigated. One study examined the effectiveness of botulinum toxin injections (BoNT-A) on function and participation by improving stroke survivors’ physical activities.

### Effectiveness of training on participation

Of the 25 articles treating social participation as a primary outcome, nine studies found positive results [22, 24, 25, 33, 37–41], while 14 studies did not show positive findings and two did not report the corresponding statistical analysis. The nine studies comprised of five RCTs and four single group pre-post study designs. The interventional protocol of the nine studies consisted of physical exercise (N = 4), supported conversation training for post-stroke aphasia (N = 1), occupational therapy (N = 1), health education (N = 1), and complex intervention (N = 2). Of the 73 studies with social participation as a secondary outcome, 26 studies had a positive result, and in these studies, the majority of intervention programs were physical exercise (N = 17). The rest studies focused on occupational therapy, self-management programs, complex intervention, health education, and invasive injection. Given the variety in included studies and difficulty in synthesis, the preliminary effectiveness of identified intervention modalities was evaluated by the number of studies that had a positive result. In summary, the preliminary effectiveness of intervention modalities for post-stroke social participation was potentially positive. Specifically, the exercise-based interventions and complex intervention seemed to be more promising than other interventions. But the effectiveness of exercise-based interventions on enhancing social participation among stroke survivors was paradoxical. The effect of self-management interventions and complex interventions needs to be further clarified given the controversial results in studies where social participation was primary outcome and secondary outcome.

## Discussion

This scoping review was performed to map the literature regarding the implementation of interventions to improve social participation following stroke and to identify the preliminary effectiveness for stroke survivors. We identified 98 studies that explored interventions for improving social participation after stroke, typically using a RCT or pilot study design.

Our scoping review reveals several different patterns of intervention. Firstly, exercise-based intervention was frequently mentioned regardless of the outcome type of social participation. It is believed that physical recovery was an important approach to enhance patients’ abilities to participate in society [6], which is consistent with the ICF framework. A previous study found that exercised-based intervention might be effective in promoting post-stroke social participation [48]. A critical concern to existing evidence is that physical exercise is a broad concept and may be implemented in diverse ways, making it hard to figure out which exercise patterns are the most effective [49]. However, it’s notable that a comprehensive and personalized exercise-based intervention may be more beneficial to orient rehabilitation modality to patients’ goals due to the diverse physical dysfunction after stroke. Considering the long-term process of stroke rehabilitation, exercise-based intervention should be conducted persistently to promote the transformation of stroke patients’ participation from family to society.

Next, occupational therapy is another key intervention pattern. Its’s expected that occupational therapy may enhance social participation following stroke by the improvement of activity level according to ICF [50]. Occupational therapy was increasingly prevalent in view of the advantages of client-centered task training and being capable of being performed in the context of their living environment. Originally activities trained were home-based ADL that people carried out to maintain well-being [51]. Recently the range of activities is being extended to leisure activities and social activities. Occupational therapist directly helps stroke patients reintegrate into society by acquiring special strategies rather than learning ability of ADL [52]. To strength the effectiveness of occupational therapy, these strategies are quite important, such as motivational enhancement and problem-solving. Furthermore, caregivers should be included into the treatment team because they might play an essential role in both assisting stroke survivors in performing activities and acting as a therapist at the time of occupational therapist’ visit interval [29].

The next modality of intervention is self-management program. When the irreversible impairment caused by the stroke occurs, stroke survivors have to learn coping skills for re-obtain a better social participation. Self-management program is considered as a key way to address the gap because it is beneficial to empower clients and strengthen their beliefs in completing a specific task [53, 54]. Therefore, it is believed that self-management program is an indispensable way to compensate for missing physical function by enhancing strategies and skills to manage individual condition and achieve social participation. However, the effect of self-management programs needs to be further verified in the future.

Complex intervention is another significant intervention modality, which may provide multidimensional supports when stroke survivors encounter low self-efficacy, inappropriate coping style, inadequate family support, and inaccessibility of social services. The essence of a complex intervention is to target several factors important for influencing social participation. For example, Mayo et al. developed a community-based complex intervention according to the research priorities, including exercise-based programs, and project-based activities promoting learning, leisure, and social activities [55]. Generally, complex intervention was mainly applied in community and participants’ homes, aiming at fully utilizing the relevant resources. Health Education used to work as a separate intervention but now is incorporated into other intervention patterns, such as complex intervention. Stroke survivors’ knowledge may facilitate the management of their own life and social participation. Other intervention modalities were reported less frequently, but may be beneficial to improve social participation, such as CRCATT, a patient-mediated question prompt list [35]. Further studies are needed to examine the effectiveness of the novel intervention pattern.

With regards to the preliminary effectiveness, our findings support a potentially positive effect on improving social participation following stroke. Furthermore, it seems that exercise-based intervention has more benefits for stroke survivors compared with non-exercise-based intervention. Of note, controversial conclusion at the same intervention pattern is common. For example, virtual reality-based limb exercise, occupational therapy, and self-management program, and complex intervention produced both positive and negative results. It is not clear whether such a mix effect is induced by the intervention itself, the different dosage, or participants’ characteristics. Therefore, the real effectiveness of the different interventions remains to be explored.

Another critical question worth our profound meditation is that how to push these intervention patterns on stroke survivors so that they can obtain sustained benefits given the long-term process of stroke rehabilitation. Some studies demonstrated a positive short-term result (assessed at post-intervention time) but a negative long-term result (assessed at follow-up time, usually three months after the end of intervention), which revealed the intervention effect faded as the supports ended. On the one hand, it is appropriate to integrate the behavior change skills into the intervention to make active participation in society a habit [56]. On the other hand, it is necessary to make the supportive resources always accessible for stroke survivors. Mobile phone is expected to become an effective tool to continue to interact with stroke survivors and their caregivers and provide various supports so as to promote them to integrate into society.

The stroke patients included into this scoping review mainly covered middle-aged and elderly stroke survivors. To the best of our knowledge, age is the high risk of stroke, and people aged over 50 show susceptibility to stroke. In fact, stroke survivors tend to be younger recently and the loss of social participation seems to be more catastrophic for young people [57]. Thus, further research is needed to identify the characteristics of social participation in young stroke survivors and to explore intervention protocols targeted at them. Our findings regarding multiple measurement tools and operational definitions of social participation are consistent with previous literature [8]. The most frequently used measures in our study were the participation subscale of SIS (SIS-P), followed by RNLI and CIQ. These differences regarding social participation measurements are influenced by inconsistent conceptualization and may further affect the findings of included studies and the comparison of results. Representation of clear and consistent conceptualization and measurement have always been an issue in social participation research [58]. Moreover, with the development of subjective social participation, a notable concern is how to improve participation experiences and feelings among stroke survivors, given the quality of be socially participating is far more important than the amount to participate for people with disability [59]. The various training dosages may be another factor leading to inconsistent results of intervention protocols. For example, the self-management program with 18 two-hour, group-based sessions, twice per week for 8 weeks showed a positive effect on post-stroke social participation [60], while the self-management protocol with 12 weekly group sessions, each lasting 2.5 h demonstrated a negative result [61]. More intensive interventions may show a better interventional effect on social participation. Furthermore, our scoping review provides referenced training period (8 weeks), sessions (10–12 sessions), duration (30 min), and frequency (2 to 3 times per week) for better improving social participation. Additionally, the lack of heterogeneity of the countries and religion where the studies were performed could compromise adaptability and application of intervention modalities of social participation. Among the intervention protocols reviewed in this study, face-to-face interventions were dominated. In the future, the effects of smartphone-based tele-intervention can be further explored, with the advantage of achieving across-culture interaction [62].

### Limitation and implication

There are a few limitations that should be acknowledged in the present scoping review. Firstly, despite every effort we have tried, three studies were excluded for unavailable full-text, which may have contributed to the incomplete synthesis of data. Secondly, we have made efforts to search eligible studies by checking references and contacting the investigators to reduce effect of incomplete data synthesis. Nevertheless, there were still some studies that mixed stroke survivors with other patients (e.g., traumatic brain injury, spinal injury, or impaired neurological conditions) being removed due to the failure to obtain the raw data of stroke survivors, which may also have an impact on the synthesis. Thirdly, there was a wide variation in the included studies, especially in the training programs, training dosages, and measurement tools of social participation, which may result in challenge in interpreting the results. While we have identified several intervention modalities, the preliminary effectiveness of current interventions on social participation among stroke survivors is hard to conclude in this scoping review. In the future, a meta-analysis may be carried out to determine the consistent discussion of some interventional protocols, such as self-management programs and occupational therapy. Finally, social participation was not widely used as a primary outcome, influencing the assessment and establishment of effective interventions. Our study distinguished the results in social participation as primary outcome and secondary outcome. More high-quality and large-sample researches are needed to identify the effectiveness of various interventions on post-stroke social participation that is viewed as a primary outcome. Additionally, the synthesis of data in our scoping review may provide some insight into participants, helpful measuring instruments, potential training protocol, and appropriate training dosage.

## Conclusion

Exercised-based intervention, occupational therapy, self-management program, and complex intervention were important intervention modalities for improvement of social participation among stroke survivors. The preliminary effectiveness of identified intervention patterns was potentially positive. High-quality researches should be conducted to a consistent discussion targeted at achieving meaningful social participation after stroke.

## Electronic supplementary material

Below is the link to the electronic supplementary material.

Supplementary Material 1

## Data Availability

All data generated or analysed during this study are included in this published article and its supplementary information files.
