# Supplementary material for: Intervention patterns and preliminary effectiveness on Social Participation following stroke: a scoping review
Source: BMC Neurol. 2023 Jul 18;23:275. doi: 10.1186/s12883-023-03250-2 (PMC10354954; doi:10.1186/s12883-023-03250-2)
Supplement: Supplementary file 1 — Supplementary Material 1 [file 12883_2023_3250_MOESM1_ESM.docx]

**Supplementary Table 1 Search strategy**

| Database | Search terms |
| --- | --- |
| Cochrane Library | (Stroke or Strokes OR Apoplexy OR Apoplexia OR Apoplectic OR Cerebrovascular accident OR Cerebrovascular accidents OR Cerebrovascular Apoplexy OR Brain Vascular Accident OR Cerebral infarction OR Brain infarction OR Brain Infarct OR Cerebral thrombosis OR Cerebral embolism OR Cerebral hemorrhage OR Encephalorrhagia OR Hematencephalon OR Subarachnoid hemorrhage OR Brain hemorrhage) in Title Abstract Keyword AND (Social participation OR Social involvement OR Social integration OR Social reintegration OR Social engagement OR Social inclusion OR Social connect OR Community participation OR Community involvement OR Community integration OR Community reintegration OR Community engagement OR Community inclusion OR Community connect OR Participation outcome OR Work Engagement) in Title Abstract Keyword |
| Web of science | #1: TS=(Stroke or Strokes OR Apoplexy OR Apoplexia OR Apoplectic OR Cerebrovascular accident OR Cerebrovascular accidents OR Cerebrovascular Apoplexy OR Brain Vascular Accident OR Cerebral infarction OR Brain infarction OR Brain Infarct OR Cerebral thrombosis OR Cerebral embolism OR Cerebral hemorrhage OR Encephalorrhagia OR Hematencephalon OR Subarachnoid hemorrhage OR Brain hemorrhage) |
|  | #2: TS=(Social participation OR Social involvement OR Social integration OR Social reintegration OR Social engagement OR Social inclusion OR Social connect OR Community participation OR Community involvement OR Community integration OR Community reintegration OR Community engagement OR Community inclusion OR Community connect OR Participation outcome OR Work Engagement) |
|  | #3: #1 AND #2 |
| Medline | #1 : (Stroke or Strokes OR Apoplexy OR Apoplexia OR Apoplectic OR Cerebrovascular accident OR Cerebrovascular accidents OR Cerebrovascular Apoplexy OR Brain Vascular Accident OR Cerebral infarction OR Brain infarction OR Brain Infarct OR Cerebral thrombosis OR Cerebral embolism OR Cerebral hemorrhage OR Encephalorrhagia OR Hematencephalon OR Subarachnoid hemorrhage OR Brain hemorrhage).mp. [mp=title, abstract, original title, name of substance word, subject heading word, floating sub-heading word, keyword heading word, organism supplementary concept word, protocol supplementary concept word, rare disease supplementary concept word, unique identifier, synonyms] |
|  | #2: (Social participation OR Social involvement OR Social integration OR Social reintegration OR Social engagement OR Social inclusion OR Social connect OR Community participation OR Community involvement OR Community integration OR Community reintegration OR Community engagement OR Community inclusion OR Community connect OR Participation outcome OR Work Engagement). mp. [mp=title, abstract, original title, name of substance word, subject heading word, floating sub-heading word, keyword heading word, organism supplementary concept word, protocol supplementary concept word, rare disease supplementary concept word, unique identifier, synonyms] |
|  | #3: #1 AND #2 |
| Pubmed | ("Stroke"[Title/Abstract] OR "Strokes"[Title/Abstract] OR "Apoplexy"[Title/Abstract] OR "Apoplexia"[Title/Abstract] OR "Apoplectic"[Title/Abstract] OR "cerebrovascular accident"[Title/Abstract] OR "cerebrovascular accidents"[Title/Abstract] OR "cerebrovascular apoplexy"[Title/Abstract] OR "brain vascular accident"[Title/Abstract] OR "cerebral infarction"[Title/Abstract] OR "brain infarction"[Title/Abstract] OR "brain infarct"[Title/Abstract] OR "cerebral thrombosis"[Title/Abstract] OR "cerebral embolism"[Title/Abstract] OR "cerebral hemorrhage"[Title/Abstract] OR "Encephalorrhagia"[Title/Abstract] OR "Hematencephalon"[Title/Abstract] OR "subarachnoid hemorrhage"[Title/Abstract] OR "brain hemorrhage"[Title/Abstract]) AND ("social participation"[Title/Abstract] OR "social involvement"[Title/Abstract] OR "social integration"[Title/Abstract] OR "social reintegration"[Title/Abstract] OR "social engagement"[Title/Abstract] OR "social inclusion"[Title/Abstract] OR (("social behavior"[MeSH Terms] OR ("Social"[All Fields] AND "behavior"[All Fields]) OR "social behavior"[All Fields] OR "sociality"[All Fields] OR "Social"[All Fields] OR "socialisation"[All Fields] OR "socialization"[MeSH Terms] OR "socialization"[All Fields] OR "socialise"[All Fields] OR "socialised"[All Fields] OR "socialising"[All Fields] OR "socialities"[All Fields] OR "socializations"[All Fields] OR "socialize"[All Fields] OR "socialized"[All Fields] OR "socializers"[All Fields] OR "socializes"[All Fields] OR "socializing"[All Fields] OR "socially"[All Fields] OR "socials"[All Fields]) AND "connect"[Title/Abstract]) OR "community participation"[Title/Abstract] OR "community involvement"[Title/Abstract] OR "community integration"[Title/Abstract] OR "community reintegration"[Title/Abstract] OR "community engagement"[Title/Abstract] OR "community inclusion"[Title/Abstract] OR "community connect"[Title/Abstract] OR "participation outcome"[Title/Abstract] OR "work engagement"[Title/Abstract]) |
| EMBASE | #1 : (Stroke or Strokes OR Apoplexy OR Apoplexia OR Apoplectic OR Cerebrovascular accident OR Cerebrovascular accidents OR Cerebrovascular Apoplexy OR Brain Vascular Accident OR Cerebral infarction OR Brain infarction OR Brain Infarct OR Cerebral thrombosis OR Cerebral embolism OR Cerebral hemorrhage OR Encephalorrhagia OR Hematencephalon OR Subarachnoid hemorrhage OR Brain hemorrhage).mp. [mp=title, abstract, heading word, drug trade name, original name, keyword, floating subheading word, candidate term word] |
|  | #2: (Social participation OR Social involvement OR Social integration OR Social reintegration OR Social engagement OR Social inclusion OR Social connect OR Community participation OR Community involvement OR Community integration OR Community reintegration OR Community engagement OR Community inclusion OR Community connect OR Participation outcome OR Work Engagement). mp. [mp= title, abstract, heading word, drug trade name, original name, keyword, floating subheading word, candidate term word] |
|  | #3: #1 AND #2 |
| CHNAHL plus | #1 : Stroke or Strokes OR Apoplexy OR Apoplexia OR Apoplectic OR Cerebrovascular accident OR Cerebrovascular accidents OR Cerebrovascular Apoplexy OR Brain Vascular Accident OR Cerebral infarction OR Brain infarction OR Brain Infarct OR Cerebral thrombosis |
|  | #2: Cerebral embolism OR Cerebral hemorrhage OR Encephalorrhagia OR Hematencephalon OR Subarachnoid hemorrhage OR Brain hemorrhage |
|  | #3: #1 OR #2 |
|  | #4: Social participation OR Social involvement OR Social integration OR Social reintegration OR Social engagement OR Social inclusion OR Social connect OR Community participation OR Community involvement OR Community integration OR Community reintegration OR Community engagement |
|  | #5: Community inclusion OR Community connect OR Participation outcome OR Work Engagement |
|  | #6: #4 OR #5 |
|  | #7: #3 AND #6 |
| PsycINFO | #1 : Stroke or Strokes OR Apoplexy OR Apoplexia OR Apoplectic OR Cerebrovascular accident OR Cerebrovascular accidents OR Cerebrovascular Apoplexy OR Brain Vascular Accident OR Cerebral infarction OR Brain infarction OR Brain Infarct OR Cerebral thrombosis |
|  | #2: Cerebral embolism OR Cerebral hemorrhage OR Encephalorrhagia OR Hematencephalon OR Subarachnoid hemorrhage OR Brain hemorrhage |
|  | #3: #1 OR #2 |
|  | #4: Social participation OR Social involvement OR Social integration OR Social reintegration OR Social engagement OR Social inclusion OR Social connect OR Community participation OR Community involvement OR Community integration OR Community reintegration OR Community engagement |
|  | #5: Community inclusion OR Community connect OR Participation outcome OR Work Engagement |
|  | #6: #4 OR #5 |
|  | #7: #3 AND #6 |
| Scopus | TITLE-ABS-KEY (Stroke or Strokes OR Apoplexy OR Apoplexia OR Apoplectic OR Cerebrovascular accident OR Cerebrovascular accidents OR Cerebrovascular Apoplexy OR Brain Vascular Accident OR Cerebral infarction OR Brain infarction OR Brain Infarct OR Cerebral thrombosis OR Cerebral embolism OR Cerebral hemorrhage OR Encephalorrhagia OR Hematencephalon OR Subarachnoid hemorrhage OR Brain hemorrhage) AND TITLE-ABS-KEY (Social participation OR Social involvement OR Social integration OR Social reintegration OR Social engagement OR Social inclusion OR Social connect OR Community participation OR Community involvement OR Community integration OR Community reintegration OR Community engagement OR Community inclusion OR Community connect OR Participation outcome OR Work Engagement) |

**Supplementary Table 2 Basic Characteristics of Included Studies**

| Studies | Year | Country | Age [M (SD) or Median (range/IQR)] | Time after onset | Comorbidities | Study Design | Sample size (T/C) | Measurement Time | Measurement Tool | Operational definition |
| --- | --- | --- | --- | --- | --- | --- | --- | --- | --- | --- |
| Mead et al. [1] | 2022 | UK | T: 67.3 (12.5);  C: 66.1 (14.3) | From 3 months to 2 years | Not clear | RCT | 76 (39/37) | T1, T2, T3 (2 months post intervention) | SIS-P | Participation |
| Avelino et al. [2] | 2021 | Brazil | T: 69 (14); C: 68 (13) | ＞6 months and ＜5 years | Hemiparesis | RCT | 50 (25/25) | T1, T2, T3 (4 weeks post intervention) | SIS-P | Participation |
| Chen et al. [3] | 2021 | China | T: 46.20 (7.02); C: 48.60 (9.95) | from 2  weeks to 6 months | Right hemisphere | Pilot RCT | 20 (10/10) | T1, T2 | WHODAS 2.0 | Social participation |
| Adamit et al. [4] | 2021 | Israel | T: 64.6 (8.2);  C: 64.4 (10.8) | ＜36 months | mild stroke severity | RCT | 66 (33/33) | T1, T2, T3 (3 months post intervention) | RNLI | Participation and independence |
| Chiu et al. [5] | 2021 | China | T: 70.83 (6.51);  C: 65.36 (16.74) | Not reported | Modified Rankin Scale score of 2–4 points | RCT | 26 (12/14) | T1, T2 | SIS-P | Participation |
| Vluggen et al. [6] | 2021 | Netherlands | T: 78.9 (7.0)  C: 79.0 (6.5) | Not reported | Not reported | RCT | 190 (99/91) | T1, T3 (6-month follow-up) | IPA | Social participation |
| Yeh et al. [7] | 2021 | China | T: 53.05 (14.53);  C: 57.36 (12.17); C: 60.17 (12.13) | ≥6 months | Cognitive impairment | RCT | 56 (20/18/18) | T1, T2 | CIQ | Social participation |
| Kringle et al. [8] | 2020 | America | 70.8 (10.9) | From 6 months to 5 years | Not clear | Pre-post-test design | 21 | T1, T2, T3 (8 weeks post intervention) | SIS-P | Participation |
| Park et al. [9] | 2020 | Korea | T: 54.9 (10.7);  C: 53.9 (16.7) | > 3 months | Upper extremity  dysfunction | RCT | 19 (10/9) | T1, Middle intervention, T2, T3 (4 weeks post intervention) | SIS-P | Social participation |
| Verberne et al. [10] | 2020 | Netherlands | T: 67.1 (10.0)  C: 71.1 (12.9) | Not reported | Not reported | Control study | 293(87/206) | T1, 6 month, 12 month | USER-P | Social participation |
| Tavares Aguiar et al. [11] | 2020 | Brazil | T: 52 (11); C: 48 (10) | ＞6 months | Upper or lower motor impairment | RCT | 22(11/11) | T1, T2, T3 (4 weeks post intervention) | SIS-P | Participation |
| Swank et al. [12] | 2020 | USA | T: 61.2 (16.9); C: 61.3 (15.2) | ＜6 months | Upper or lower motor impairment | RCT | 73(37/36) | T1, T2, T3 (3 months post intervention) | SIS-P | Participation |
| Esmaeili et al. [13] | 2020 | Canada | T: 58.0 (6.7); C: 57.5 (18.0) | > 6 months | Reduced dynamic balance abilities | RCT | 18 | T1, T2, T3 (6 weeks post intervention) | RNLI | Community integration |
| Aprileet al. [14] | 2020 | Italy | T: 69.5 (10.9); C: 68.5 (11.5) | 2 weeks to 6 months | Upper extremity impairment | RCT | 224(111/113) | T1, T2, T3 (3 months post intervention) | SF-36 | Participation |
| Yeh et al. [15] | 2019 | China | T: 50.63 (3.99); C: 60.21 (3.10) | ≥6 months | Cognitive impairment | RCT | 30(15/15) | T1, T2 | CIQ | Social participation |
| Wolf et al. [16] | 2019 | USA | T: 61.6 (10.2); C: 58.8 (11.7) | ≥1 months | Unmet functional goals | RCT | 44(24/20) | T1, T2, T3 (9 months post intervention) | SIS-P | Participation |
| Warland et al. [17 | 2018 | UK | 58 (7.1) | ≥12 weeks | Upper limb function impairment | Pre-post-test design | 12 | T1, T2, T3 (4 weeks post intervention) | SIPSO | Participation |
| Song et al. [18] | 2019 | Korea | T: 62.20 (14.74); C: 61.42 (14.80) | ≥6 months | Upper and lower function impairment | RCT | 49(25/24) | T1, T2 | CIQ | Community integration and social role limitations |
| Liu et al. [19] | 2019 | China | T: 60.47 (5.61); C: 60.46 (5.91) | 1 to 6 years | Balance impairment | RCT | 89(45/44) | T1, T2, T3 (3 and 12 months post intervention) | CIQ | Community integration |
| Dehem et al. [20] | 2019 | Belgium | T: 67.3 (11.1); C: 68.6 (19.1) | < 1 month since stroke | Upper extremity function impairment | RCT | 45(23/22) | T1, T2, T3 (6 months post stroke) | SIS-P | Social participation |
| Aprile et al. [21] | 2019 | Italy | T: 56.43 (12.93); C: 61.58 (9.00) | 2 weeks to 6 months | Lower extremity function | Pre-post-test design | 26(14/12) | T1, T2 | WHS | Participation |
| Amatya et al. [22] | 2019 | Australia | 56.5 (15.2) | ≤10 years | Upper or lower limb function impairment | Pre-post-test design | 35 | T1, T3 (6 and 12 weeks post intervention) | The Euro-Quality of life | Participation |
| Mansfield et al. [23] | 2018 | Canada | T: 66 (17); C: 67 (13) | ＞6 months | Balance impairment | RCT | 83(41/42) | T1, T2 | SIPSO | Social integration |
| Lewthwaite et al. [24] | 2018 | USA | 61 | 14-106 days | Upper-extremity motor impairment | RCT | 361(119/120/122) | T1, T2, T3 (6 months and 1 year after randomization) | RNLI; SIS-P | Participation |
| Karthikbabu et al. [25] | 2018 | India | T1: 57.2 (11.5); T2: 54 (14.1); C: 54.8 (12.5) | ≥6 months | Poor trunk performance | RCT | 108(36/36/36) | T1, T2, T3 (3 and 12 months post intervention) | RNLI | Community reintegration |
| Escher et al. [26] | 2018 | USA | 54.47 (11) | ＞6 months | Aphasia | Pre-post-test design | 19 | T0 (4 weeks before intervention), T1, T2, T3 (3 months post intervention) | SIS-P | Participation |
| Baer et al. [27] | 2017 | UK | T: 71.23 (12.52); C: 74.50 (11.7) | ＜3 months | Lower limb function impairment | RCT | 77(38/39) | T1, T2, T3 (6 months after randomization) | SIS | Participation |
| Preston et al. [28] | 2017 | Australia | 68 (12) | Not reported | Lower limb function impairment | Pre-post-test study | 20 | T1, T2, T3 (3 months post intervention) | IPA | Participation |
| Poulin et al. [29] | 2016 | Canada | 49.25 (11.35) | ≤12 months | Executive function impairment | Partially RCT | 9(5/4) | T1, T2, T3 (1 month post intervention) | LIFE-H | Social participation |
| Kootker et al. [30] | 2016 | Netherlands | T: 61 (45, 79); C: 61 (25, 76) | ≥3 months | Mild cognitive impairment | RCT | 61(31/30) | T1, T2, T3 (4 and 8 months post intervention) | USER-P | Social participation |
| Shin et al. [31] | 2016 | Korea | T: 57.2 (10.3); C: 59.8 (13.0) | Not reported | Unilateral upper extremity functional deficits | RCT | 46(24/22) | T1, Middle intervention, T2, T3 (1 month post intervention) | SIS-P | Social participation |
| Sandberg et al. [32] | 2016 | Sweden | T: 71.3 (7.0); C: 70.4 (8.1) | ＞3 days | Not clear | RCT | 56(29/27) | T1, T2, T3 (6 months after randomization) | SIS-P | Participation |
| Raghavan et al. [33] | 2016 | USA | 52 (14) | ≥6 months | Upper limb function impairment | Pre-post-test design | 13 | T1, T2, T3 (1 year post intervention) | SIS-P | Participation |
| Liao et al. [34] | 2016 | China | T1: 60.8 (8.3); T2: 62.9 (10.2); C: 59.8 (9.1) | ≥6 months） | Lower limb function impairment | RCT | 84(28/28/28) | T1, T2 | FAI | Participation in daily activities |
| Faria et al. [35] | 2016 | Portugal | T: 58 (48, 71); C: 53(50.5, 65.5) | Not reported | Cognitive function impairment | RCT | 18(9/9) | T1, T2 | SIS-P | Social participation |
| Chua et al. [36] | 2016 | UK | T: 62.1 (10.3); C: 60.7 (10.7) | ≤8 weeks | Lower limb function impairment | RCT | 106(53/53) | T1, Middle intervention, T2, T3 (4, 16, and 40 weeks post intervention) | SIS-P | Participation |
| Schmid et al. [37] | 2015 | USA | 64.8 (9.14) | >6 months | Not clear | Pre-post-test design | 10 | T1, T2 | IMPACT | Participation |
| Nijenhuis et al. [38] | 2015 | Netherlands | 59 (13) | 6 months to 5 years | Upper limb function impairment | Pre-post-test design | 21 | T1, T2, T3 (2 months post intervention) | SIS-P | Participation |
| Mckenna et al. [39] | 2013 | Australia | T: 62.18 (13.57); C: 67.38 (10.60) | Not reported | Not clear | RCT | 24(11/13) | T1, T2, T3 (3 months post intervention) | SIPSO | Community integration |
| McEwen et al. [40] | 2014 | Canada | T: 57.5 (14.0); C: 54.4 (14.0) | ≤3 months | No obvious complications | RCT | 35(19/16) | T1, T2, T3 (3 months after discharge) | CPI; SIS-P | Community participation; Participation |
| Mayo et al. [41] | 2015 | Canada | T: 61 (12); C: 65 (11) | ≤ 5 years | Not clear | Randomized trial | 186(93/93) | T1, 3, 6, 12 and 15 months after entry | RNLI | Participation |
| Hayward et al. [42] | 2015 | Australia | 57 | 9 months earlier | Upper- and lower-limb hemisensory loss and left-sided perceptual neglect | Case report | 1 | T1, T2, T3 (3 months post intervention) | SIS-P | Participation |
| Alabdulwahab et al. [43] | 2015 | Saudi Arabia | 45.2 (12.5) | ≥3 months | Unilateral motor deficits | Pre-post-test design | 23 | T1, T2 | SIS-P | Perceived participation |
| Ostwald et al. [44] | 2014 | America | T: 65.75 (9.26); C: 66.98 (9.04) | <12 months | Not clear | RCT | 159(79/80) | T1, T2, T3 (3, 6 and 9 months post intervention) | FIM | Positive social interaction |
| Olaleye et al. [45] | 2014 | Nigeria | At: 60.6 (10.2) | Not reported | Upper or lower limb function impairment | Pre-post-test design | 25 | T1, T2 | RNLI | Community reintegration |
| Marzolini et al. [46] | 2014 | Canada | 63.86 (12.0) | >3 months | Upper or lower limb function impairment | Pre-post-test design | 120 | T1, T2 | Stroke impact survey | Participation |
| Immink et al. [47] | 2014 | Australia | T: 56.1 (13.6); C: 63.2 (17.4) | >9 months | Hemiparesis | RCT | 22(11/11) | T1, T2 | SIS-P | Social Participation |
| Tamplin et al. [48] | 2013 | Australia | 58.0 (13.8) | Not reported | Aphasia | Pre-post-test design | 13 | T1, 12 weeks and 20 weeks after entry | Subscales of SIS | Communication |
| Serena et al. [49] | 2013 | China | T: 54.96 (15.30); C: 56.6 (14.967) | Not reported | Hemiplegia | RCT | 50(25/25) | T1, T2, T3 (time was not reported) | CIQ | Community integration |
| Nadeau et al. [50] | 2013 | America | T1: 60.1 (12.3); T2: 62.6 (13.3); C: 63.3 (12.5) | <45 days | Lower limb function impairment | RCT | 408(139/126/143) | T1, T2 | SIS-P | Participation |
| Mayo et al. [51] | 2013 | Canada | T: 67.7 (14.4); C: 67.8 (12.3) | <12 months | Lower limb function impairment | RCT | 87(43/44) | T1, 1, 3, 6, 9, and 12 months after entry | SIS-P; Subscales of RAND-36 | Social and role participation |
| Annie et al. [52] | 2013 | Canada | T: 62.5 (12.5); C: 63.2 (12.4) | Not reported | Not clear | RCT | 186(94/92) | T1, T2, T3 (6 months post intervention) | LIFE-H | Participation |
| Ada et al. [53] | 2013 | Australia | T1: 70 (11); T2: 64 (12); C: 63 (13) | Not reported | Lower limb function impairment) | RCT | 102(34/34/34) | T1, 2, 4, 6, and 12 months after entry | Adelaide Activities Profile | Community participation |
| Shaughnessy et al. [54] | 2012 | America | 64.3 (9.2) | Not reported | Hemiparetic gait deficits | RCT | 63(29/34) | T1, T2 | SIS-P | Social activities |
| Lund et al. [55] | 2012 | Norway | T: 75 (7.2); C: 79 (6.5) | >3 months | No obvious complications | RCT | 86(39/47) | T1, T2 | SF-36 | Social participation |
| Flansbjer et al. [56] | 2012 | Sweden | 66 (4) | Not reported | Lower limb function impairment | RCT | 18(11/7) | T1, T2, T3 (5 months and 4 years post intervention) | SIS-P | Perceived participation |
| Chumbler et al. [57] | 2012 | America | T 67.1 (9.5); C: 67.7 (10.0) | <24 months | Not clear | RCT | 48(25/23) | T1, T2, T3 (3 months post intervention) | LLFDI | Social role frequency |
| Nnacy et al. [58] | 2011 | Canada | T: 70 (14); C: 72 (13) | Not reported | Mobility problem | RCT | 190(96/94) | T2, T3 (6 months post intervention) | IMPACT | Role participation |
| Markle-Reid et al. [59] | 2011 | Canada | T: 75.8 (12.4); C: 70.6 (14.5) | <18 months | Depressed, cognitively impaired | RCT | 82(43/39) | T1, T2 | RNLI | Community reintegration |
| Egan et al. [60] | 2010 | Canada | 68.6 (6.4) | <2 months | Not clear | Pre-post-test design | 41 | T1, T2 | RNLI | Community reintegration |
| Jones et al. [61] | 2009 | England | 61.5 (8.15) | Not reported | Not clear | Pre-post-test design | 10 | T1, T2 | SIPSO | Participation |
| Lord et al. [62] | 2008 | New Zealand | T: 64.2 (14.8); C: 60.7 (17.6) | Not reported | Gait impairment | RCT | 30(16/14) | T1, T2, T3 (6 months post intervention) | SIPSO | Participation and social integration |
| Huijbregts et al. [63] | 2008 | Canada | T: 68.0 (10.5); C: 71.0 (7.6) | >3 months | Arm or leg impairment | Longitudinal cohort design | 30(18/12) | T1, T2, T3 (3 months post intervention) | RNLI | Reintegration to normal Living |
| Carol et al. [64] | 2008 | Canada | 65.88 (6.73) | >6 months | Not clear | Pre-post-test design | 17 | T1, T2, T3 (8 months post intervention) | LDB | Perceived leisure competence |
| Wu et al. [655] | 2007 | China | T: 71.44 (6.42); C: 71.94 (6.79) | Not reported | Upper-extremity movement impairment | RCT | 26(13/13) | T1, T2 | SIS-P | Participation performance |
| Kendall et al. [66] | 2007 | Australia | 65.96 (10.67) | Not reported | Not clear | RCT | 100(58/42) | T1, T2, T3 (6 months post intervention) | Subscale of SS-QOL | Social roles, family roles, and work productivity |
| Egan et al. [67] | 2007 | Canada | T: 75.7 (10.2); C: 65.6 (7.6) | >6 months | Not clear | RCT | 14(6/8) | T1, T2 | RNLI | Participation |
| Desrosiers et al. [68] | 2007 | Canada | T: 72.4 (12.1); C: 70.8 (10.8) | >5 years | Not clear | RCT | 56(29/27) | T1, T2 | Active leisure score | Participation in leisure |
| Chan et al. [69] | 2006 | Canada | T: 53.8 (15.4); C: 54.4 (13.7) | <12month | Not clear | RCT | 52(26/26) | T1, 2, 4 and 6 weeks after entry | CIQ | Home integration, social integration, and productive activity |
| Thorsen et al. [70] | 2005 | Sweden | T:71; C:72 | 5 to 7 days | Mild to moderate disability | RCT | 54(30/24) | T1, T2, T3 (6 months, 12 months and 5 years post intervention) | Katz ADL Index; FAI | Participation |
| Studenski et al. [71] | 2005 | America | T: 69.5 (10.3); C: 74.0 (10.4) | 3 to 28 days | Mild to moderate disability | RCT | 93(44/49) | T1, T2, T3 (6 months post intervention) | SIS-P | Social participation |
| Andersen et al. [72] | 2002 | Denmark | T1: 69.8 (9.9); T2: 74.1 (11.4); C: 68.3 (12.3) | Not reported | Motor capacity impaired | RCT | 155(54/53/48) | 6 months after entry | FAI | Leisure and social activities |
| Friedemann et al. [73] | 2001 | England | T: 55.4 (10.9); C: 53.9 (7.4) | Not reported | Aphasia | RCT | 17(10/7) | T1, T2 | CAL | Amount and quality of communication |

Note: C, Control group; T, Trail group; RCT, Randomized Controlled Trial; T1, Baseline; T2, Post-intervention; T3, Follow-up; CAL, The Communicative Activity Log; CIQ, The Community Integration Questionnaire; CPI, The Community Performance Indicators; FAI, The Frenchay Activity Index; LDB, The Leisure Diagnostic Battery; LIFE-H, The Assessment of Life Habits; LLFDI, The Disability component scale of Late-Life Function and Disability Instrument; IMPACT, The International Classification of Functioning (ICF) (Disability and Health) Measure of Participation and Activities; IPA, The Impact on Participation and Autonomy Questionnaire; RAND-36, the RAND-36 Item Health Survey; RNLI, Reintegration to Normal Living Index; SF-36, Short Form Health Survey; SIPSO, Subjective Index of Physical and Social Outcome; SIS-P, Participation subscale of Stroke Impact Scale; SS-QOL, Stroke Specific Quality of Life Scale; USER-P, Utrecht Scale for Evaluation of Rehabilitation-Participation; WHS, The Walking Handicap Scale

**Supplementary** **Table 3**  **Intervention Information of Included Studies (secondary outcome)**

| **Studies** | **Setting** | **Dosage** | **Experiment Group** | **Control Group** | **Results** |
| --- | --- | --- | --- | --- | --- |
| Mead et al. [1] | Three Scottish stroke services | For 6 weeks | Six telephone calls: session 1: introduction and psychoeducation about fatigue; session 2: goal setting and activity planning; session 3: progress assessment and goal modification; session 4: cognitive restructuring; session 5: dealing with blocks and setbacks, session 6: overview and future plans, booster session: review progress and make further plans | Did not receive allocated intervention | Negative |
| Avelino et al. [2] | Home and community | — | The provision of a cane for a month | received a booklet containing how to perform  stretching exercises of the lower-limb muscles | Negative |
| Chen et al. [3] | Department of Rehabilitation Medicine | 45-min training daily, 5 days/week, for 4 weeks | Robot-assisted arm training (RAT) | General cognitive and occupational rehabilitation dedicated for unilateral spatial neglect | Positive |
| Adamit et al. [4] | A community based Health Care Service | 10 weekly sessions | The Functional and Cognitive Occupational Therapy (FaCoT) intervention: OT taught and practiced the use of cognitive (Initiation, Inhibition, Planning and Decision-Making) and behavioral (Self-perception, Situation, interpretation and Future prediction) strategies | Standard care: receive the same cognitive-functional assessments | Positive |
| Chiu et al. [5] | T: Home  C: Hospital setting | Once a week for 6 weeks | The home-reablement group received goal-directed ADL training: the occupational therapist taught the ADL tasks and provided implementation strategies, such as task analysis, task redesign, and work simplification | 30 minutes of physical therapy and 30 minutes of occupational therapy twice a week | Negative |
| Vluggen et al. [6] | Geriatric rehabilitation stroke units and primary care | 2 to 6 months | An integrated multidisciplinary geriatric rehabilitation programme: inpatient neurorehabilitation, home-based self-management training, and stroke education | Usual Care | Negative |
| Yeh et al. [7] | Hospitals | 60 min/day, 3 days/week, for a total of 12 weeks | Sequential combination of aerobic exercise and cognitive training | An aerobic exercise training group (progressive resistive stationary bicycle training14); a cognitive training group (computer-based cognitive training) | Negative |
| Kringle et al. [8] | Community | 12 one-on-one sessions, 3 times per week for 4 weeks | Sedentary behavior education, self-monitoring of sitting time, self-assessment of successes and challenges, problem solving, and scheduling of future activities | NA | Negative |
| Park et al. [9] | Rehabilitation hospital | 20 sessions of 30-min robotic intervention  (5 days/week, 4 weeks) | Passive robotic intervention group | Active‑assistive robotic intervention group | Positive |
| Verberne et al. [10] | Not reported | Not reported | Nurse-led stroke aftercare: consultation and necessary aftercare | Care-as-usual | Negative |
| Tavares Aguiar et al. [11] | Not reported | Three 40-minute session per week over 12 weeks | Aerobic-treadmill training | Outdoor-overground walking | Positive |
| Swank et al. [12] | Inpatient rehabilitation facility | Two 30-minute activity bouts each day | Patient-Directed Activity Program (PDAP) and three hours of therapy daily: upper and lower extremity activities based on principles of neuroplasticity and self-management | Usual care only: three hours of therapy daily | Negative |
| Esmaeili et al. [13] | Not reported | Nine training sessions over 3 weeks | Treadmill training with intense and unpredictable perturbations | Treadmill walking-only training | Positive |
| Aprileet al. [14] | Rehabilitation centers | At least 25 rehabilitation sessions | Upper Limb Robotic Rehabilitation | Conventional rehabilitation | Positive |
| Yeh et al. [15] | Hospital-based rehabilitation units | 36 training sessions: 60 minutes of training sessions 2 or 3 times per week for 12 to 18 weeks | Aerobic Exercise and Computer-Based Cognitive Training | Nonaerobic exercise and unstructured mental activities | Negative |
| Wolf et al. [16] | Outpatient occupational therapy | 12, 45-minute sessions of occupational therapy, one to two times per week | An evidence-based, patient-centered, TST-based rehabilitation protocol augmented with metacognitive strategy use | Task-Specific training (TST) | Negative |
| Warland et al. [17 | Not reported | Nine, 40-minute exercise sessions, 3 days per week over 3 weeks | An adapted version of a commercially available, virtual-reality gaming system (the Personalized Stroke Therapy system) | NA | Positive |
| Song et al. [18] | Not reported | 30-minute session per day, 5 times a week for 4 weeks | Cognitive orientation to daily occupational performance (CO-OP): cognitive strategy of goal-plan-do check and motor-based tasks in the repetitive action | Task-specific upper extremity training | Negative |
| Liu et al. [19] | Neurorehabilitation laboratory | 90-minute interventions 2 days per week for 8 weeks | Cognitive behavior therapy and task-oriented balance training (TOBT): enhance the subjects’ balance efficacy using 2 main strategies, namely cognitive restructuring and behavioral modification | General health education and task-oriented balance training (TOBT) | Negative |
| Dehem et al. [20] | Inpatient rehabilitation centres | 4 sessions per week over 9 weeks | Upper-limb robotic-assisted therapy (RAT) and conventional therapy | Conventional therapy | Positive |
| Aprile et al. [21] | Rehabilitation centers | 3 times a week, 20 sessions | End-effector Robot-Assisted Gait Training | Conventional gait training (CG) | Negative |
| Amatya et al. [22] | Neurorehabilitation unit | Not reported | Botulinum toxin injections (BoNT-A) | NA | Positive |
| Mansfield et al. [23] | Academic hospitals | Two 1-hour training sessions per week for 6 weeks and two 1-hour ‘booster’ training | Perturbation-based balance training | Traditional balance training | Short-term result: negative Long-term result: positive |
| Lewthwaite et al. [24] | Outpatient setting | T and C1: 30 one-hour, treatment sessions across 16 weeks; C2: an average of 11.2 total hours over (range 0-46 hours) 16 weeks | A patient-centered Accelerated Skill Acquisition Program: capacity building, skill acquisition (movement skills and self-direction skills), motivation enhancement, autonomy support | Group 1: usual occupational therapy (DEUCC) Group 2: usual therapy (UCC) | Positive |
| Karthikbabu et al. [25] | Outpatient stroke units | One-hour exercise session, 3 sessions a week over a duration of 6 weeks | T1: Plinth trunk exercise regimes T2: Swiss ball-based trunk exercise regimes | Standard physiotherapy | Positive |
| Escher et al. [26] | Not reported | 6 hours per day, 5 days per week for 4 weeks | A community-based, occupational therapy intervention situated within an intensive comprehensive aphasia program (ICAP) | NA | Negative |
| Baer et al. [27] | Hospital-based stroke units | At least 3 sessions per week for 8 weeks | Treadmill training and normal gait re-education | Normal gait re-education | Negative |
| Preston et al. [28] | Home | Five 60-min sessions over 3 months | Self-management program | NA | Positive |
| Poulin et al. [29] | Home | 16 one-hour sessions, twice a week, for eight weeks | Occupation-based strategy training using an adapted version of the Cognitive Orientation to daily Occupational Performance approach: combines several key elements: participant-chosen goals, use of a global problem-solving strategy, dynamic performance analysis, guided discovery and domain-specific strategy application | Computer-based EF training | Negative |
| Kootker et al. [30] | Ambulatory rehabilitation settings | 13-16 sessions across 4 months, each session lasting about 1 hour | Individually tailored cognitive behavioral therapy (CBT) | Computerized cognitive training | Negative |
| Shin et al. [31] | Rehabilitation Center of an urban rehabilitation hospital | 20 sessions for 30 min per day, across 4 weeks | Smart Glove (SG): Virtual reality (VR)-based rehabilitation | Conventional intervention group | Positive |
| Sandberg et al. [32] | Ambulatory care | Twice weekly 60-minute for 12 weeks | Twice-Weekly Intense Aerobic Exercise | Received no organized rehabilitation or scheduled physical exercise | Negative |
| Raghavan et al. [33] | Not reported | 45-minute intervention twice a week for 6 weeks | Music Upper Limb Therapy-Integrated (MULT-I) | NA | Short-term result: negative Long-term result: positive |
| Liao et al. [34] | Research laboratory | T1: 12 sessions T2: 18 sessions | T1: low-intensity whole-body vibration (WBV) and various leg exercises; T2: high-intensity whole-body vibration (WBV) and various leg exercises | Various leg exercises | Negative |
| Faria et al. [35] | Not reported | Twelve 20-min sessions, distributed from 4 to 6 weeks | Virtual reality based cognitive rehabilitation (Reh@City) | Conventional rehabilitation | Negative |
| Chua et al. [36] | A Community hospital | 45 minutes of physiotherapy 6 times per week for 8 weeks | Electromechanical gait trainers and conventional physiotherapy | Conventional physiotherapy | Negative |
| Schmid et al. [37] | Not reported | Six 2-hour sessions, once per week for the first five sessions, the last session performed 3 weeks later | Group Occupational Therapy for Falls | NA | Negative |
| Nijenhuis et al. [38] | Home | 30 minutes per day, six days per week for 6 weeks | Self-administered training using an arm and hand device with motivational gaming environment | NA | Negative |
| Mckenna et al. [39] | Not reported | One hour per week over six weeks | The Bridges stroke self-management program and usual stroke rehabilitation | Usual rehabilitation only | Short-term result: positive Long-term result: negative |
| McEwen et al. [40] | Rehabilitation centers | 45-minute session, twice per week, the total number of sessions varies (≤10) | The Cognitive Orientation to daily Occupational Performance (CO-OP) | Usual outpatient rehabilitation | Negative |
| Mayo et al. [41] | Eleven community sites | Twice a week for three hours each time for three blocks, each lasting three months | Group 1: community-based program Group 2: wait-list community-based program | NA | Negative |
| Hayward et al. [42] | Home | 5 days a week for 4 weeks, for a total of 20 sessions | Self-Administered, Home-Based SMART (Sensorimotor Active Rehabilitation Training) | NA | Not tested |
| Alabdulwahab et al. [43] | Outpatient rehabilitation unit | One-hour duration three times per week for four weeks | Functional limb overloading (FLO) | Limb Overloading Resistance Training | Positive |
| Ostwald et al. [44] | Home | Monthly personalized letters for 12 months | Mailed information programs | Home-based (HB) intervention | Positive |
| Olaleye et al. [45] | Primary health center | 45-60 minutes session, twice weekly for 10 consecutive weeks | Physiotherapy intervention in primary health center (PHCG): consists of exercises to strengthen the extremities, improve balance, enhance walking ability and encourage arm and hand function | NA | Negative |
| Marzolini et al. [46] | Home | 90-minute exercise class, once per week for 6 months | Cardiac Rehabilitation Exercise Program (CRPs) | NA | Positive |
| Immink et al. [47] | Recreation room | 90-minute group classes, once per week and 40-minute individual home practice sessions every day | Yoga intervention | No intervention | Negative |
| Tamplin et al. [48] | Not reported | 2-hour rehearsal weekly (90 minutes of singing songs and simple vocal exercises and a 30-minute coffee break for rest and socialization） | Choir intervention | NA | Not tested |
| Serena et al. [49] | Local rehabilitation hospital | Not reported | Occupational Lifestyle Redesign Programme (OLSR) and conventional OT programme | Conventional OT programme | Positive |
| Nadeau et al. [50] | Rehabilitation center | 90-minute sessions, 3 times per week for 12-16 weeks | Group 1: locomotor training Group2: home exercise program (HEP) | Usual Care | Negative |
| Mayo et al. [51] | Home | 13 30-minute intervention over 1 year | Complex intervention: exercise and project-based activities promoting learning, leisure, and social activities, done as individuals and in groups | Task-oriented exercise and walking | Positive |
| Annie et al. [52] | Not reported | Weekly for the first 2 months, biweekly during the third month, and monthly for the past 3 months | Proactive series of calls from trained hospital staff (WE CALL) | Standard of care initiated by patients (YOU CALL) | Negative |
| Ada et al. [53] | Not reported | 30 min train, 3 times per week for 16 weeks for T1 and eight-weeks for T2 | T1: four-month treadmill walking program T2: two-month treadmill walking program | No intervention | Negative |
| Shaughnessy et al. [54] | Not reported | Three 40-minute exercise sessions weekly over 6-month | Task-oriented treadmill training program | Stretching program | Negative |
| Lund et al. [55] | Senior centres | 72 sessions over 9 months, once a week for 2 hours | Lifestyle Redesign (lifestyle course and physical activity) | Physical activity only | Negative |
| Flansbjer et al. [56] | Not reported | Twice weekly for 10 weeks | Progressive resistance training | Usual daily activities | Negative |
| Chumbler et al. [57] | Home | three 1-hour home visits every 12 to 16 days within 5 weeks, weekly monitor daily use of an in-home messaging device and 5 telephone intervention calls every 14 days over 3 months | Multifaceted stroke telerehabilitation (STeleR) intervention: 3 1-hour home visits (televisits), by a trained assistant to assess physical performance and help communicate the instruction of exercises and use of assistive technology and/or adaptive techniques recommended by a licensed teletherapist; participants’daily use of an in-home messaging device (IHMD) that was monitored weekly by the teletherapist; and 5 telephone intervention  calls | Usual care | Negative |
| Nnacy et al. [58] | Acute-care hospitals and home | 3 to 30 contacts over 6 weeks | Complex intervention: physical, emotional and psychological impairments, role participation restrictions and health perception | Usual care | Negative |
| Markle-Reid et al. [59] | Home | 12 months | Specialized interprofessional team approach: home care service, continuity of care provider, stroke risk and community reintegration assessment tools, stroke prevention, rehabilitation, and community reintegration strategies, access to home care services, mechanisms for team community and collaboration, information systems | Usual home care services | Negative |
| Egan et al. [60] | Community | 1 to 8 times within 4-month | Community Stroke Navigation service | NA | Negative |
| Jones et al. [61] | Community | Not reported | self-management workbook intervention | NA | Negative |
| Lord et al. [62] | Hospitals and community | Twice a week for 7 weeks | Assistant-led, community-based physiotherapy: balance activities, open and closed-chain exercises, practice of selective components of the gait cycle, walking tasks and treadmill training | Hospital-based, outpatient, physiotherapy | Negative |
| Huijbregts et al. [63] | Rehabilitation center | 17 two-hour sessions, twice per week for 8 weeks, and a booster session 6 weeks later | Self-management program with land and water exercise (Moving On after Stroke or MOST) | Standard education program (Living with Stroke or LWS) | Positive |
| Carol et al. [64] | Aphasia Centre of Ottawa | 4-hour intervention, 2 times per week for 8 weeks | Multidisciplinary Family Centered Community Stroke (FCCS) program: consisted of couple therapy, recreation therapy/leisure education, physiotherapy/exercise therapy, speech therapy, recreation participation and informal peer contact | NA | Negative |
| Wu et al. [655] | Rehabilitation departments | 2 hours per day on 10-15 consecutive weekdays; Restraint: 6 hours per day for 2 to 3weeks | Modified constraint-induced movement therapy (mCIMT) | Traditional rehabilitation | Negative |
| Kendall et al. [66] | Community | 2 hours course per week across 6-week period | Chronic Disease Self-Management (CDSM) course and standard post-discharge rehabilitation | Standard post-discharge rehabilitation | Negative |
| Egan et al. [67] | Community | Over 2 to 4 months | Community-based occupational therapy | Usual care | Negative |
| Desrosiers et al. [68] | Home or community | 60 minutes per session, once a week for 8 to 12 weeks | Home Leisure Education Program: leisure awareness, self-awareness, competency development | Visited by the recreational therapist but the topics discussed were unrelated to leisure | Positive |
| Chan et al. [69] | Outpatient rehabilitation center | Three 2-h sessions each week for 6 weeks | Motor relearning programme | Conventional therapy programme | Positive |
| Thorsen et al. [70] | Home | 14 weeks | A home rehabilitation group (HRG) | Conventional rehabilitation group (CRG) | Negative |
| Studenski et al. [71] | Home | 36 sessions over 12 weeks | Therapeutic Exercise | Usual care | Positive |
| Andersen et al. [72] | Home | T1: three 1-hour home visits (at 2, 6 and 12 weeks after discharge) T2: 1-8 times 1-hour home visits | Group1: Follow-up home visits by a physician (INT1-HVP) Group2: Physiotherapist instruction in the patient’s home (INT2-PI) | Standard aftercare | Negative |
| Friedemann et al. [73] | Hospital | 3 hours per day over 10 days | Constraint-Induced Therapy (CI) | Conventional aphasia therapy | Positive |

Reference

1. Mead G, Gillespie D, Barber M, House A, Lewis S, Ensor H, et al. Post stroke intervention trial in fatigue (POSITIF): Randomised multicentre feasibility trial. Clinical rehabilitation. 2022;36(12):1578-89.
2. Avelino PR, Nascimento LR, Ada L, de Menezes KKP, Teixeira-Salmela LF. Using a cane for one month does not improve walking or social participation in chronic stroke: An attention-controlled randomized trial. Clinical rehabilitation. 2021;35(11):1590-8.
3. Chen ZJ, Gu MH, He C, Xiong CH, Xu J, Huang XL. Robot-Assisted Arm Training in Stroke Individuals With Unilateral Spatial Neglect: A Pilot Study. Frontiers in neurology. 2021;12:691444.
4. Adamit T, Shames J, Rand D. Effectiveness of the Functional and Cognitive Occupational Therapy (FaC(o)T) Intervention for Improving Daily Functioning and Participation of Individuals with Mild Stroke: A Randomized Controlled Trial. International journal of environmental research and public health. 2021;18(15).
5. Chiu EC, Chi FC, Chen PT. Investigation of the home-reablement program on rehabilitation outcomes for people with stroke: A pilot study. Medicine. 2021;100(26):e26515.
6. Vluggen T, van Haastregt JCM, Tan FE, Verbunt JA, van Heugten CM, Schols J. Effectiveness of an integrated multidisciplinary geriatric rehabilitation programme for older persons with stroke: a multicentre randomised controlled trial. BMC geriatrics. 2021;21(1):134.
7. Yeh TT, Chang KC, Wu CY, Chen CJ, Chuang IC. Clinical efficacy of aerobic exercise combined with computer-based cognitive training in stroke: a multicenter randomized controlled trial. Topics in stroke rehabilitation. 2022;29(4):255-64.
8. Kringle EA, Terhorst L, Gibbs BB, Campbell G, McCue M, Skidmore ER. Activating Behavior to Reduce Sedentary Behavior After Stroke: A Nonrandomized Pilot Feasibility Study. The American journal of occupational therapy : official publication of the American Occupational Therapy Association. 2020;74(6):7406205030p1-p10.
9. Park JH, Park G, Kim HY, Lee JY, Ham Y, Hwang D, et al. A comparison of the effects and usability of two exoskeletal robots with and without robotic actuation for upper extremity rehabilitation among patients with stroke: a single-blinded randomised controlled pilot study. Journal of neuroengineering and rehabilitation. 2020;17(1):137.
10. Verberne DPJ, van Mastrigt G, Ponds R, van Heugten CM, Kroese M. Economic evaluation of nurse-led stroke aftercare addressing long-term psychosocial outcome: a comparison to care-as-usual. BMJ open. 2021;11(2):e039201.
11. Tavares Aguiar L, Nadeau S, Rodrigues Britto R, Fuscaldi Teixeira-Salmel L, Caetano Martins J, Ribeiro Samora GA, et al. Effects of aerobic training on physical activity in people with stroke: A randomized controlled trial. NeuroRehabilitation 2020; 46 (3):391-401.
12. Swank C, Trammell M, Callender L, Bennett M, Patterson K, Gillespie J, et al. The impact of a patient-directed activity program on functional outcomes and activity participation after stroke during inpatient rehabilitation-a randomized controlled trial. Clin Rehabil 2020;34(4):504-14.
13. Esmaeili V, Juneau A, Dyer J-O, Lamontagne A, Kairy D, Bouyer L, et al. Intense and unpredictable perturbations during gait training improve dynamic balance abilities in chronic hemiparetic individuals: a randomized controlled pilot trial. J Neuroeng Rehabil 2020;17(1):1-13.
14. Aprile I, Germanotta M, Cruciani A, Loreti S, Pecchioli C, Cecchi F, et al. Upper Limb Robotic Rehabilitation After Stroke: A Multicenter, Randomized Clinical Trial. J Neurol Phys Ther 2020, 44 (1):3-14.
15. Yeh T-t, Chang K-c, Wu C-y. The Active Ingredient of Cognitive Restoration: A Multicenter Randomized Controlled Trial of Sequential Combination of Aerobic Exercise and Computer-Based Cognitive Training in Stroke Survivors With Cognitive Decline. Arch Phys Med Rehabil 2019;100(5):821-7.
16. Wolf TJ, Doherty M, Boone A, Rios J, Polatajko H, Baum C, et al. Cognitive oriented strategy training augmented rehabilitation (COSTAR) for ischemic stroke: a pilot exploratory randomized controlled study. Disabil Rehabil 2021;43(2):201-210.
17. Warland A, Paraskevopoulos I, Tsekleves E, Ryan J, Nowicky A, Griscti J, et al. The feasibility, acceptability and preliminary efficacy of a low-cost, virtual-reality based, upper-limb stroke rehabilitation device: a mixed methods study. Disabil Rehabil 2019;41(18):2119-34.
18. Song C-S, Lee O-N, Woo H-S. Cognitive strategy on upper extremity function for stroke: A randomized controlled trials. Restor Neurol Neuros 2019;37(1):61-70.
19. Liu T-W, Ng GYF, Chung RCK, Ng SSM. Decreasing Fear of Falling in Chronic Stroke Survivors Through Cognitive Behavior Therapy and Task-Oriented Training. Stroke 2019; 50(1):148-54.
20. Dehem S, Gilliaux M, Stoquart G, Detrembleur C, Jacquemin G, Palumbo S, et al. Effectiveness of upper-limb robotic-assisted therapy in the early rehabilitation phase after stroke: A single-blind, randomised, controlled trial. Ann Phys Rehabil Med 2019;62(5):313-20.
21. Aprile I, Iacovelli C, Goffredo M, Cruciani A, Galli M, Simbolotti C, et al. Efficacy of end-effector Robot-Assisted Gait Training in subacute stroke patients: Clinical and gait outcomes from a pilot bi-centre study. Neurorehabilitation 2019;45(2):201-12.
22. Amatya B, Lizama LEC, Elmalik A, Bastani A, Galea MP, Khan F. Multidimensional evaluation of changes in limb function following botulinum toxin injection in persons with stroke. Neurorehabilitation 2019;45(1):67-78.
23. Mansfield A, Aqui A, Danells CJ, Knorr S, Centen A, DePaul VG, et al. Does perturbation-based balance training prevent falls among individuals with chronic stroke? A randomised controlled trial. BMJ open 2018;8(8):e021510.
24. Lewthwaite R, Winstein CJ, Lane CJ, Blanton S, Wagenheim BR, Nelsen MA, et al. Accelerating Stroke Recovery: Body Structures and Functions, Activities, Participation, and Quality of Life Outcomes From a Large Rehabilitation Trial. Neurorehab Neural Re 2018;32(2):150-65.
25. Karthikbabu S, Chakrapani M, Ganesan S, Ellajosyula R, Solomon JM. Efficacy of Trunk Regimes on Balance, Mobility, Physical Function, and Community Reintegration in Chronic Stroke: A Parallel-Group Randomized Trial. J Stroke Cerebrovasc Dis 2018;27(4):1003-11.
26. Escher AA, Amlani AM, Viani AM, Berger S. Occupational Therapy in an Intensive Comprehensive Aphasia Program: Performance and Satisfaction Outcomes. Am J Occup Ther 2018;72(3):7203205110p1-p7.
27. Baer GD, Salisbury LG, Smith MT, Pitman J, Dennis M. Treadmill training to improve mobility for people with sub-acute stroke: a phase II feasibility randomized controlled trial. Clin Rehabil 2018;32(2):201-12.
28. Preston E, Dean CM, Ada L, Stanton R, Brauer S, Kuys S, et al. Promoting physical activity after stroke via self-management: a feasibility study. Top Stroke Rehabil 2017; 24(5):353-60.
29. Poulin V, Korner-Bitensky N, Bherer L, Lussier M, Dawson DR. Comparison of two cognitive interventions for adults experiencing executive dysfunction post-stroke: a pilot study. Disabil Rehabil 2017;39(1):1-13.
30. Kootker JA, Rasquin SMC, Lem FC, van Heugten CM, Fasotti L, Geurts ACH. Augmented Cognitive Behavioral Therapy for Poststroke Depressive Symptoms: A Randomized Controlled Trial. Arch Phys Med Rehab 2017;98(4):687-94.
31. Shin J-H, Kim M-Y, Lee J-Y, Jeon Y-J, Kim S, Lee S, et al. Effects of virtual reality-based rehabilitation on distal upper extremity function and health-related quality of life: a single-blinded, randomized controlled trial. J Neuroeng Rehabil 2016;13:17.
32. Sandberg K, Kleist M, Falk L, Enthoven P. Effects of Twice-Weekly Intense Aerobic Exercise in Early Subacute Stroke: A Randomized Controlled Trial. Arch Phys Med Rehab 2016;97(8):1244-53.
33. Raghavan P, Geller D, Guerrero N, Aluru V, Eimicke JP, Teresi JA, et al. Music Upper Limb Therapy-Integrated: An Enriched Collaborative Approach for Stroke Rehabilitation. Front Hum Neurosci 2016;10:498.
34. Liao Lin-Rong, Ng GYF, Jones AYM, Mei-Zhen H, Pang MYC. Whole-Body Vibration Intensities in Chronic Stroke: A Randomized Controlled Trial. Med Sci Sport Exer 2016;48(7):1227-38.
35. Faria AL, Andrade A, Soares L, SB IB. Benefits of virtual reality based cognitive rehabilitation through simulated activities of daily living: a randomized controlled trial with stroke patients. J Neuroeng Rehabil 2016;13(1):96.
36. Chua J, Culpan J, Menon E. Efficacy of an Electromechanical Gait Trainer Poststroke in Singapore: A Randomized Controlled Trial. Arch Phys Med Rehab 2016;97(5):683-90.
37. Schmid AA, Miller KK, Van Puymbroeck M, DeBaun-Sprague E, Shively C, Peterson E, et al. Feasibility and results of a pilot study of group occupational therapy for fall risk management after stroke. Brit J Occup Ther 2015;78(10):653-60.
38. Nijenhuis SM, Prange GB, Amirabdollahian F, Sale P, Infarinato F, Nasr N, et al. Feasibility study into self-administered training at home using an arm and hand device with motivational gaming environment in chronic stroke. J Neuroeng Rehabil 2015;12:89.
39. McKenna S, Jones F, Glenfield P, Lennon S. Bridges self-management program for people with stroke in the community: A feasibility randomized controlled trial. Int J Stroke 2015;10(5):697-704.
40. McEwen S, Polatajko H, Baum C, Rios J, Cirone D, Doherty M, et al. Combined Cognitive-Strategy and Task-Specific Training Improve Transfer to Untrained Activities in Subacute Stroke: An Exploratory Randomized Controlled Trial. Neurorehab Neural Re 2015;29(6):526-36.
41. Mayo NE, Anderson S, Barclay R, Cameron JI, Desrosiers J, Eng JJ, et al. Getting on with the rest of your life following stroke: a randomized trial of a complex intervention aimed at enhancing life participation post stroke. Clin Rehabil. 2015;29(12):1198-211.
42. Hayward KS, Neibling BA, Barker RN. Self-Administered, Home-Based SMART (Sensorimotor Active Rehabilitation Training) Arm Training: A Single-Case Report. Am J Occup Ther 2015;69(4):6904210020p1-8.
43. Alabdulwahab SS, Ahmad F, Singh H. Effects of Functional Limb Overloading on Symmetrical Weight Bearing, Walking Speed, Perceived Mobility, and Community Participation among Patients with Chronic Stroke. Rehabil Res Pract 2015;2015:241519.
44. Ostwald SK, Godwin KM, Cron SG, Kelley CP, Hersch G, Davis S. Home-based psychoeducational and mailed information programs for stroke-caregiving dyads post-discharge: a randomized trial. Disabil Rehabil 2014;36(1):55-62.
45. Olaleye OA, Hamzat TK, Owolabi MO. Stroke rehabilitation: should physiotherapy intervention be provided at a primary health care centre or the patients' place of domicile? Disabil Rehabil 2014;36(1):49-54.
46. [64] Marzolini S, Tang A, McIlroy W, Oh PI, Brooks D. Outcomes in people after stroke attending an adapted cardiac rehabilitation exercise program: does time from stroke make a difference? J Stroke Cerebrovasc Dis 2014;23(6):1648-56.
47. Immink MA, Hillier S, Petkov J. Randomized controlled trial of yoga for chronic poststroke hemiparesis: motor function, mental health, and quality of life outcomes. Top Stroke Rehabil 2014;21(3):256-71.
48. Tamplin J, Baker FA, Jones B, Way A, Lee S. 'Stroke a Chord': the effect of singing in a community choir on mood and social engagement for people living with aphasia following a stroke. NeuroRehabilitation 2013;32(4):929-41.
49. Serena S. W. Ng, Chan DYL, Chan MKL, Chow KKY. Long-term Efficacy of Occupational Lifestyle Redesign Programme for Strokes. Hong Kong J Occup Th 2013;23(2):46-53.
50. Nadeau SE, Wu SS, Dobkin BH, Azen SP, Rose DK, Tilson JK, et al. Effects of task-specific and impairment-based training compared with usual care on functional walking ability after inpatient stroke rehabilitation: LEAPS Trial. Neurorehab Neural Re 2013;27(4):370-80.
51. Mayo NE, MacKay-Lyons MJ, Scott SC, Moriello C, Brophy J. A randomized trial of two home-based exercise programmes to improve functional walking post-stroke. Clin Rehabil. 2013;27(7):659-71.
52. Annie Rochette, Korner-Bitensky N, Bishop D, Teasell R, White CL, Bravo G, et al. The YOU CALL-WE CALL Randomized Clinical Trial Impact of a Multimodal Support Intervention After a Mild Stroke. Circ-Cardiovasc Qual 2013;6(6):674-9.
53. Ada L, Dean CM, Lindley R. Randomized trial of treadmill training to improve walking in community-dwelling people after stroke: the AMBULATE trial. Int J Stroke 2013;8(6):436-44.
54. Shaughnessy M, Michael K, Resnick B. Impact of Treadmill Exercise on Efficacy Expectations, Physical Activity, and Stroke Recovery. J Neurosci Nurs 2012;44(1):27-35.
55. Lund A, Michelet M, Sandvik L, Wyller TB, Sveen U. A lifestyle intervention as supplement to a physical activity programme in rehabilitation after stroke: a randomized controlled trial. Clin Rehabil 2012;26(6):502-12.
56. Flansbjer UB, Lexell J, Brogardh C. Long-term benefits of progressive resistance training in chronic stroke: a 4-year follow-up. J Rehabil Med 2012;44(3):218-21.
57. Chumbler NR, Quigley P, Li X, Morey M, Rose D, Sanford J, et al. Effects of telerehabilitation on physical function and disability for stroke patients: a randomized, controlled trial. Stroke 2012;43(8):2168-74.
58. Nancy E.Mayo, Scott S. Evaluating a complex intervention with a single outcome may not be a good idea: an example from a randomised trial of stroke case management. Age Ageing 2011;40(6):718-24.
59. Markle-Reid M, Orridge C, Weir R, Browne G, Gafni A, Lewis M, et al. Interprofessional stroke rehabilitation for stroke survivors using home care. Can J Neurol Sci 2011;38(2):317-34.
60. Egan M, Anderson S, McTaggart J. Community navigation for stroke survivors and their care partners: description and evaluation. Top Stroke Rehabil 2010;17(3):183-90.
61. Jones F, Mandy A, Partridge C. Changing self-efficacy in individuals following a first time stroke: preliminary study of a novel self-management intervention. Clin Rehabil. 2009;23(6):522-33.
62. Lord S, McPherson KM, McNaughton HK, Rochester L, Weatherall M. How feasible is the attainment of community ambulation after stroke? A pilot randomized controlled trial to evaluate community-based physiotherapy in subacute stroke. Clin Rehabil 2008;22(3):215-25.
63. Huijbregts MPJ, Myers AM, Streiner D, Teasell R. Implementation, Process, and Preliminary Outcome Evaluation of Two Community Programs for Persons with Stroke and Their Care Partners. Top Stroke Rehabil 2008;15(5):503-20.
64. Carol, Ryan CA, Stiell KM, Gailey GF, Makinen JA. Evaluating a family centered approach to leisure education and community reintegration following a stroke. Ther Recreation J 2008;42(2):119-31.
65. Wu CY, Chen CL, Tsai WC, Lin KC, Chou SH. A randomized controlled trial of modified constraint-induced movement therapy for elderly stroke survivors: changes in motor impairment, daily functioning, and quality of life. Arch Phys Med Rehab 2007;88(3):273-8.
66. Kendall E, Catalano T, Kuipers P, Posner N, Buys N, Charker J. Recovery following stroke: the role of self-management education. Soc Sci Med 2007;64(3):735-46.
67. Egan M, Kessler D, Laporte L, Metcalfe V, Carter M. A pilot randomized controlled trial of community-based occupational therapy in late stroke rehabilitation. Top Stroke Rehabil 2007;14(5):37-45.
68. Desrosiers J, Noreau L, Rochette A, Carbonneau H, Fontaine L, Viscogliosi C, et al. Effect of a home leisure education program after stroke: A Randomized controlled trial. Arch Phys Med Rehab 2007;88(9):1095-100.
69. Chan DY, Chan CC, Au DK. Motor relearning programme for stroke patients: a randomized controlled trial. Clin rehabil 2006;20(3):191-200.
70. Thorsen AM, Holmqvist LW, de Pedro-Cuesta J, von Koch L. A randomized controlled trial of early supported discharge and continued rehabilitation at home after stroke - Five-year follow-up of patient outcome. Stroke 2005;36(2):297-302.
71. Studenski S, Duncan PW, Perera S, Reker D, Lai SM, Richards L. Daily functioning and quality of life in a randomized controlled trial of therapeutic exercise for subacute stroke survivors. Stroke 2005;36(8):1764-70.
72. Andersen HE, Eriksen K, Brown A, Schultz-Larsen K, Forchhammer BH. Follow-up services for stroke survivors after hospital discharge--a randomized control study. Clin rehabil. 2002;16(6):593-603.
73. Friedemann Pulvermüller, Neininger B, Elbert T, Mohr B, Rockstroh B, Koebbel P, et al. Constraint-Induced Therapy of Chronic Aphasia After Stroke. Stroke 2001;32(7):1621-6.

**Supplementary Table 4 Cochrane risk of bias in included randomized controlled trials**

| **Studies** | Random sequence generation | Allocation concealment | Blindness of participant and personnel | Blindness of outcome assessment | Incomplete outcome data | Selective reporting | Other bias |
| --- | --- | --- | --- | --- | --- | --- | --- |
| de Rooij et al. [23] | U | L | H | L | L | L | L |
| Tarrant et al. [24] | L | L | H | L | L | L | L |
| Harel-Katz et al. [26] | L | U | H | L | L | L | L |
| Hedman et al. [29] | L | L | H | L | L | L | L |
| Stark et al. [31] | L | H | H | L | L | L | L |
| Brouwer et al. [33] | L | L | H | L | L | L | L |
| Van de Ven et al. [34] | L | U | H | U | L | L | L |
| Kessler et al. [35] | L | L | H | L | L | L | L |
| Wang et al. [36] | L | L | H | L | L | L | L |
| Tielemans et al. [37] | L | L | H | U | L | L | L |
| Mckellar et al. [38] | L | L | H | U | L | L | L |
| Guidetti et al. [39] | L | U | H | L | L | L | L |
| Muller et al. [40] | L | L | H | U | L | L | L |
| Kim et al. [41] | L | L | H | L | L | L | L |
| Marsden et al. [42] | L | L | H | L | L | L | L |
| Harrington et al. [43] | L | L | H | L | L | L | L |
| Smith et al. [44] | L | L | H | U | L | L | L |
| Katz-Leurer et al. [45] | L | U | H | L | L | L | L |
| Parker et al. [46] | L | L | H | L | L | L | L |

Note: L, Low risk; H, High risk; U, Unclear risk.

**Supplementary Table 5 Quality assessment results for non-randomized controlled trials**

| Studies | 1 | 2 | 3 | 4 | 5 | 6 | 7 | 8 | 9 |
| --- | --- | --- | --- | --- | --- | --- | --- | --- | --- |
| Bin Zainal et al. [25] | Y | Y | NA | N | Y | Y | Y | Y | Y |
| Cruice et al. [27] | Y | Y | NA | N | Y | Y | Y | Y | Y |
| Chinchai et al. [28] | Y | Y | NA | N | Y | Y | Y | Y | Y |
| Aramaki et al. [30] | Y | Y | NA | N | Y | Y | Y | Y | Y |
| Kamwesiga et al. [32] | Y | N | Y | Y | Y | N | Y | Y | Y |
| Hinckley et al. [47] | Y | Y | Y | Y | Y | N | Y | Y | Y |

Note: 1, Clear description of “cause” and “effect”; 2, Consistent baseline; 3, Treated similarly other than the exposure or intervention of interest; 4, Control group; 5, Multiple outcome measurements; 6, Complete follow-up; 7, The same outcome measurement; 8, Reliable outcome measurement; 9: Appropriate statistical analysis; Y, Yes; N, No; U, Unclear; NA, Not applicable.
